# Novel Virtual Reality Intervention for Stress Reduction Among Patients With or at Risk for Cardiovascular Disease: Mixed Methods Pilot Study

**DOI:** 10.2196/66557

**Published:** 2025-08-06

**Authors:** Katherine E Makaroff, Christopher Van, Vincent Grospe, Lynae Edmunds, Marcella A Calfon-Press, Karol E Watson, Tamara Horwich

**Affiliations:** 1 David Geffen School of Medicine at University of California, Los Angeles, 10833 Le Conte Avenue, Los Angeles, CA, 90095, United States, 1 310 825 8816; 2 University of California, San Francisco School of Medicine, San Francisco, CA, United States

**Keywords:** heart disease risk factors, stress reduction, digital health, pilot, virtual reality, stress, risk, cardiovascular disease, CVD, behavioral cardiology, mixed methods, cardiology, cardiac rehabilitation, survey, blood pressure, heart rate

## Abstract

**Background:**

Virtual reality (VR) has emerged as a promising, low-risk strategy to manage many forms of psychological stress and may be a modality to improve cardiovascular health. Recent scientific statements on the mind-heart-body connection call for better adherence to psychological screening and adoption of more holistic “behavioral cardiology” interventions that improve the overall health of patients with or at risk for cardiovascular disease (CVD).

**Objective:**

The aim of this study is to assess safety and preliminarily explore how a VR experience can aid in stress reduction among patients with or at risk for CVD.

**Methods:**

A convergent mixed methods approach was used for this single-arm prospective pilot study. In total, 20 patients were recruited from the University of California Los Angeles adult cardiology clinics and cardiac rehabilitation. Surveys and physiologic parameters were collected before, during, and after a 30-minute VR experience aimed at relaxation. The primary outcome was the State-Trait Anxiety Inventory-State (STAI-S) scale. They participated in a 90-minute visit, during which they completed surveys, including the STAI-S scale, before and after a 30-minute VR experience. Physiological parameters were also collected before, during, and after the experience. Visits concluded with semistructured interviews analyzed with inductive thematic analysis to add depth and nuance to our analysis.

**Results:**

STAI-S scale scores after the VR experience were significantly decreased from baseline (median 31, IQR 28-38 vs median 24, IQR-29.25; *P*<.001). Verbal feedback revealed that participants experienced a relaxing sense of “distance from stress” moderated by unexpected, intense audiovisual components. Heart rate significantly decreased (mean 73, SD 8 vs mean 67, SD 6 beats per minute; *P*<.001), while blood pressure (mean systolic 128, SD 14 vs mean systolic 129, SD 18 mm Hg; *P*=.75 and mean diastolic 79, SD 9 vs mean diastolic 80, SD 10 mm Hg; *P*=.60) and galvanic skin response (mean 0.74, SD 0.89 vs mean 0.70, SD 0.57 microsiemens; *P*=.45) remained the same. Changes in heart rate variability parameters were consistent with increased vagal tone over time but were only statistically significant at certain time points. Survey results and interviews generally indicated safety, tolerability, and openness to using VR again.

**Conclusions:**

This sample of patients with CVD or risk of CVD had above-average stress, consistent with epidemiological data; the statistically and clinically significant decrease in subjective perception of stress partially converged with physiologic data. Overall, the VR intervention was a safe and feasible stress reduction method. Future research is needed to evaluate the effectiveness of this immersive therapy in reducing cardiovascular risk profiles.

## Introduction

### Background

Western medicine has traditionally treated the heart and mind as separate entities. However, emerging data point to a powerful “mind-heart-body connection” in which all are interconnected and interdependent [1-3]. Research has clearly demonstrated that positive psychological states are associated with a lower risk of cardiovascular disease (CVD) and mortality, while negative psychological factors such as chronic stress, anxiety, and depression can negatively impact cardiovascular health [1-10]. Further, chronic stress is associated with a 40%‐50% increase in the risk of coronary artery disease [4]. Studies demonstrate that persistent psychological distress, including anxiety and depression, is an independent predictor of morbidity and mortality in those with established CVD [11-13]. Data from the international case-control, also known as the INTERHEART study (effect of potentially modifiable risk factors associated with myocardial infarction in 52 countries), show that psychosocial stressors account for 33% of the risk for myocardial infarction [10,14]. Psychological health in patients with or at risk for CVD represents an opportunity for risk reduction. The American Heart Association’s 2021 Scientific Statement on psychological health, well-being, and the mind-heart-body connection called for better adherence to psychological screening measures and adoption of more holistic “behavioral cardiology” interventions that improve overall health in patients with or at risk for CVD [2].

To aid in several health care challenges, many physicians and patients are harnessing the power of emerging technologies. Virtual reality (VR) is a technology that provides an immersive experience of a computer-generated, 3D image or environment using a head-mounted display. VR has presented a low-risk way to interrupt the brain’s “default mode network” (DMN), which is a particular set of brain structures that underlie the negative mental states marked by worry, rumination, and stress [15-18]. The DMN is also responsible for the “baseline buzzing” and drive for the mind to wander or “forage” for new information even when trying to relax or think about nothing at all [18]. Further, it has been shown that people become unhappier the longer time their minds spend wandering [19]. VR has now demonstrated promise as a treatment modality for anxiety, phobias, depression, autism, and posttraumatic stress disorder as well as a way to aid in meditation [20-29]. Thus, VR is a potentially powerful tool to target stress reduction in patients with CVD.

### Aims and Research Question

The aim of this study was to determine whether stress levels could be reduced immediately after experiencing a novel VR intervention in patients sampled from the University of California Los Angeles (UCLA) cardiology clinics and cardiac rehabilitation (CR) program. This project directly addressed the “heart-mind-body” connection by using an innovative VR intervention aimed at reducing stress in those with CVD. This aim was addressed through a convergent mixed methods design: this question was examined quantitatively by changes in (1) subjective patient-reported stress levels on a validated survey, (2) blood pressure (BP), (3) galvanic skin response (GSR), (4) heart rate (HR), and (5) heart rate variability (HRV). Semistructured interviews performed after the VR experience were analyzed using inductive thematic analysis to understand participant experiences and the potential therapeutic value of the VR experience. The quantitative and qualitative data were examined in parallel and integrated to look for areas of convergence or divergence across the data.

## Methods

### Study Design

**Figure 1.**
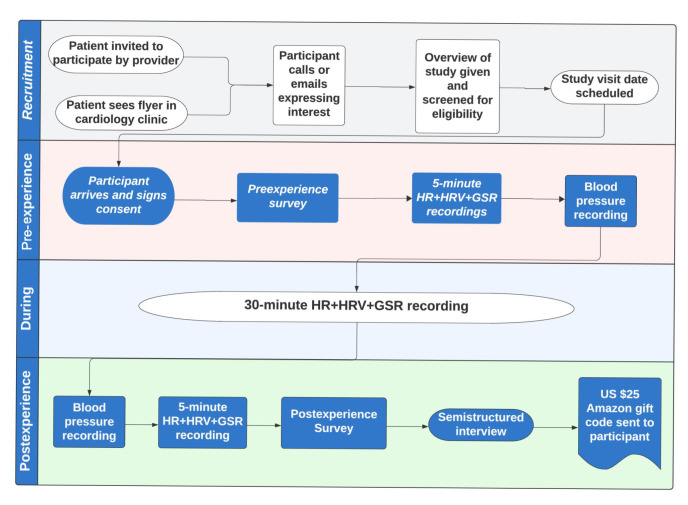
Study procedure flowchart. GSR: galvanic skin response; HR: heart rate; HRV: heart rate variability.

According to a framework proposed by a multidisciplinary group of experts for assessing immersive therapeutics [30], this study blends features of a VR1 study, which focuses on developing content in partnership with patients, and VR2 study, which tests treatment feasibility, acceptability, and tolerability. Going through this piloting phase is vital prior to designing subsequent studies comparing clinical outcomes between one group receiving the VR treatment and another receiving a control treatment (ie, VR3). A convergent mixed methods approach was used for this single-arm prospective pilot study. Figure 1 displays a study procedure flowchart.

Eligible participants were recruited to attend 1 in-person study visit lasting ~90 minutes. Participants were asked to sit and complete informed consent before proceeding to complete a pre-experience survey that took about 5 to 10 minutes to complete. It included the Perceived Stress Scale-10 (PSS-10) [31-34], 20-item State-Trait Anxiety Inventory-State scale (STAI-S) [35-40], and level of experience with various stress reduction methods. It also asked about exercise, caffeine, and sleep both generally and on the day of the study. Next, participants were connected to equipment, and a 5-minute HR and HRV recording was collected using the well-validated Polar H10 HR monitor [41,42], a commercial chest-based electrocardiogram strap considered among researchers as the standard for accurately quantifying cardiovascular metrics after multilead electrocardiograms [43-45]. The HR monitor was connected via Bluetooth to the validated third-party smartphone app EliteHRV [46] (while the patient rested quietly in a semirecumbent position). A 5-minute GSR [47-49] recording was taken simultaneously using the validated NeuLog sensor and software [50]. BP was recorded with an Omron 3 series digital BP monitor and cuff immediately before and after the VR experience (delivered through a Meta Quest 2 VR headset). During the experience, participants remained in the semirecumbent position, while HR, HRV, and GSR were measured continuously. A final 5-minute postexperience HR, HRV, and GSR recording was taken. Participants then completed the postexperience survey consisting of the STAI-S scale, Immersive Tendencies Questionnaire (ITQ) [51], Simulation Sickness Questionnaire (SSQ) [52,53], overall rating of VR intervention, level of prior experience with VR, and demographics. The study visit concluded with a 10‐ to 15-minute semistructured interview. Participants were emailed a US $25 Amazon e-gift code.

### Ethical Considerations

The study was approved by the UCLA Institutional Review Board (#21‐000705), and the ClinicalTrials.gov registry number was NCT0498465. Written informed consent was obtained. The privacy and confidentiality of research participants’ data and identity were maintained. Participants were compensated with US $25 Amazon gift cards.

### Recruitment

We recruited 20 patients from the UCLA cardiology outpatient clinics and CR program who were 18 years and older of age, English speaking (rationale for English only: surveys and consent are not translated into languages other than English and study team members are primarily English speakers, there was no funding to provide translation or medical interpreters during participation), and able to give informed consent. Patients were excluded due to the presence of conditions that interfere with VR use, including history of seizure, facial injury, significant hearing or visual impairment, individuals with dangerous unstable arrhythmias (ventricular tachycardia or fibrillation) or a myocardial infarction in the past 4 weeks, individuals in acute decompensated heart failure, those with factors known to contribute to cybersickness, including postural instability or motion sickness. Potentially eligible participants self-referred or were referred to a research coordinator by the primary investigator after an initial chart review. These patients were screened over the phone to confirm eligibility and assess interest.

### VR Experience

**Figure 2.**
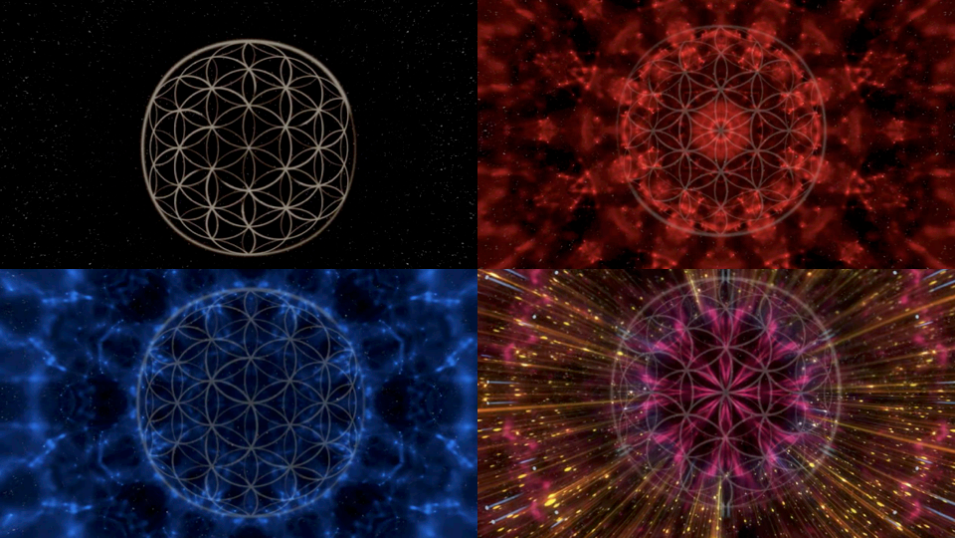
Representative images of virtual reality experience (courtesy of Harmony Media).

This 30-minute experience developed by Harmony Media Company is an immersive therapy created to lower stress and anxiety. The experience was not designed specifically for those with CVD, but rather for use in a more general population. User feedback regarding the experience was obtained informally from healthy adults at several points during development. It delivers a proprietary combination of colorful and fractal sacred geometric visual effects synchronized with a nonverbal, binaural audioscape that aims to exert its therapeutic effect by disrupting the DMN of brain signaling (Figure 2). The content is comprised of predefined (noninteractive) primary and background looping visuals, animated textures, and specific prerendered sequences allowing for predictability, measurability, and performance stability. The focus area (approximately 80% of the visual field ahead or on the horizon) is where the participants’ attention is directed (360°). Visuals are on the anterior horizon and are emitted from a central point of view at the center of the visual horizon. The participant can move their head around and continue to experience the visual effects that appear before them, but the content is not dynamic in real time nor does the content react to the participants’ movements. Color and sound changes mark subtle content transitions. Additional elements such as specific frequencies of binaural audio are incorporated to effect transitions.

### Surveys

See Multimedia Appendix 1 for full surveys. To measure the degree to which situations in one’s life are appraised as stressful, with a time recall of 1 month, the PSS-10, one of the most widely used psychological instruments for measuring perception of stress [31-34], was scored and summarized and then compared to sex, age, and race norms [34]. The primary outcome was (presurvey vs postsurvey) change in STAI-S scale (Δ-STAI-S). It is meant to measure, via self-report, the presence and severity of current symptoms of anxiety and a generalized propensity for anxiety. The State Anxiety Sub-Scale asks respondents how they feel “right now” using items that measure subjective feelings of apprehension, tension, nervousness, worry, and activation or arousal of the autonomic nervous system using a 4-point Likert scale. Scores for each scale range from 20 to 80, with higher scores indicating greater stress or anxiety (clinically significant symptoms suggested with a score of ≥39‐40).

Several studies have used the State Anxiety Scale to measure change immediately following situational stress-inducing or stress-reducing interventions, and they show that the instrument is sufficiently responsive to capture short-term changes [35-40]. To explore immersive tendencies as a predictor of responsiveness to VR, the ITQ, which is used to measure differences in individual tendencies to experience presence or immersion, was used, while the SSQ was included to evaluate the safety and tolerability of VR by measuring the frequency of acute VR discomfort (eg, headache, vertigo, nausea, and eye strain) resulting from sensory mismatch between the visual and vestibular systems called “cybersickness.” Overall rating (0 being the worst to 10 being the best) and ITQ responses were scored and analyzed using summary statistics, while the SSQ scores were interpreted using 5 standardized categories [53].

### Physiologic Data

HRV reflects complex neurocardiac regulatory interactions, including the balance of inputs from the autonomic nervous system. For HR and HRV variables, raw R-R intervals for each participant were exported from EliteHRV to Premium Kubios HRV Analysis Software (version 3.4.3; Kubios Oy). Preprocessing of the data used the automatic beat correction algorithm built into the Premium Kubios packaging [54]. The 30-minute (during VR) recording was then broken into six 5-minute periods, with each HRV variable for the period extracted for analysis. This allowed for the creation of longitudinal models across 8 time points, given that the HRV variables examined are valid with a recording period of 5 minutes [55-57]. These time points are denoted as (1) pre-experience (−5 to 0 minutes), during experience: (2) 0 to 5 minutes, (3) 6 to 10 minutes, (4) 11 to 15 minutes, (5) 16 to 20 minutes, (6) 21 to 25 minutes, (7) 26 to 30 minutes, and (8) postexperience (31 to 35 minutes). Longitudinal models provided greater granularity of analysis with potential pattern recognition across the cohort across time points.

Two HRV time-domain variables were analyzed. Root-mean-square of successive differences (RMSSD) is considered the most relevant and accurate measure of autonomic nervous system activity and specifically estimates vagal-mediated changes in HRV [56]. Standard deviation of the interbeat interval of normal sinus beats (SDNN) is another overall estimate of HRV, and in short-term recordings, mainly reflects parasympathetic input predominantly from slow-paced breathing [56]. HRV frequency domain results included the following variables (analyzed using the Fast Fourier Transformation method): (1) low frequency (LF) to high frequency (HF) ratio (LF:HF): while not exact, this measure can indicate the balance of sympathetic versus parasympathetic tone. Decreases in this score reflect increased parasympathetic (or decreased sympathetic) tone. The following absolute and relative power of the HRV frequency domains were also included: (2) very low frequency band (0.0033‐0.04 Hz): it provides insight into parasympathetic activity, (3) LF band (0.04‐0.15 Hz): this band mainly reflects baroreceptor activity during resting conditions and can reflect both sympathetic and parasympathetic tone, and (4) HF band (0.15‐0.40 Hz): this band reflects vagal tone and is referred to as the respiratory band because it is associated with variations in HR related to the respiratory cycle. Lower HF power is correlated with stress, panic, anxiety, or worry] [55-57].

GSR is another parameter that detects changes in autonomic activity through sweat gland function. Heightened psychological and physiological arousal (eg, stress) increases sympathetic activation leading to increased skin conductance detected by the GSR sensor [46].

### Statistical Analysis

Descriptive statistics of the cohort’s demographics (including experience with VR previously) were summarized along with cardiac conditions or risk factors gathered from the electronic medical record; quantitative variables were formatted and summarized using Microsoft Excel (version 16.75) with Real Statistics add-on and further analyzed with RStudio (version 2023.06.1+524; Copyright 2022 by Posit Software, PBC) using either 2-sample paired 2-tailed *t* tests or the nonparametric equivalent, Wilcoxon signed rank sum test, along with 95% CIs. A significance level of α<.05 was used. Subsequent BP, HR, HRV, and GSR data were compared to pre-experience (baseline) measurements to look for statistically significant changes across time points. A multivariable linear regression model was used to evaluate for potential survey variable predictors of the change in stress as measured by our primary outcome variable, Δ-STAI-S scale score. The effect size (*r*) for the Wilcoxon paired signed rank test was computed for Δ-STAI-S by dividing the *z*-value by the square root of the sample size (corresponding to the total number of pairs, n=20). The *r* value varies from 0 to 1, and we used the following interpretation cutoffs commonly published in the literature: 0.1 to <0.3=small effect, 0.3 to <0.5=moderate effect, and ≥0.5=large effect. To further contextualize our findings, we identified the threshold for discriminating a minimal clinically important difference as 5 units on the STAI-S scale. This is a conservative estimate based on the widely accepted half a SD (½ SD) rule of thumb for interpreting changes in health-related quality of life instruments [58-60]. For the STAI-S scale with 20 items each with a 1 to 4 scale, this creates a range from 20 to 80 or 60 points (1 SD=10 units and ½ SD=5 units). For complete physiologic and survey datasets, please see Multimedia Appendix 2.

### Qualitative Analysis

Interviews were analyzed using reflexive thematic analysis, a qualitative research method in which the researcher not only identifies themes but also reflects during the process on how their own interpretations and biases might influence the analysis [61]. A medical student conducted the interviews and analysis. Using a semistructured interview guide (Multimedia Appendix 1) with open-ended questions while maintaining a neutral and welcoming tone throughout the study visit helped to ensure that the data collected reflected the participant’s honest feedback. The same open-minded approach was taken in the analysis, using an overall social constructivist theoretical framework [62]. An inductive approach was used, and analysis sought to reflect all the participants’ actual responses rather than only code and theme development being directed by existing concepts, theories, and ideas. This allowed for a broader understanding of how the participants’ personal preferences and feedback were informed by previous experiences, knowledge, and abilities. Familiarization with the data was done by reading through the transcription multiple times with annotation for potential areas of interest or repetition of topics or concepts. A total of 11 initial codes were then generated with examples of text supporting each code being labeled and sorted under initial themes. A “critical friend,” a volunteer researcher with previous qualitative analysis experience, was consulted after coding to help talk through and clarify ideas while providing impartial feedback. They were given access to original interview transcripts so that they could make their own coding decisions. This helped to ensure the quality of analysis by challenging the primary analyst to adopt a more independent stance toward the research and ensure the analysis was coherent. The codes and supporting examples were then reviewed; themes or sub-themes were constructed and refined.

### Mixed Methods Analysis

Roughly equal weight was given to both qualitative and quantitative data while exploring the same question. Results from each data stream were compared to see if they reached the same conclusions through triangulation or in a complementary way, with qualitative data providing greater depth of understanding to quantitative results and vice versa [63].

## Results

### Recruitment and Study Population

In total, 48 individuals were referred as potential participants (provider-referred: n=37, self-referred through flyer: n=7, ClinicalTrials.gov or other: n=4). Of these, 42 were screened for eligibility via a phone call with a study staff member, 5 did not meet inclusion criteria, 17 declined participation, and 20 were enrolled or completed participation. The remaining 6 referred individuals were called by study staff but were unable to be reached. A total of 20 participants were included for all demographics or survey outcomes, while 18 included were for physiologic variables. Technical difficulties were encountered during the study visit for participant 17, leading to a lack of physiologic data. Data from participant 10 were consistently found to be a statistical outlier, likely due to the fact that they were a heart transplant recipient. Participant demographics, cardiac conditions or risk factors, and other comorbidities are shown in Table 1. The mean age of participants was 66 (SD 15; range 27-80) years. There were 9 female and 11 male participants. The most common stress relief activity was exercise, followed by deep breathing and then meditation (Multimedia Appendix 1).

**Table 1.** Participant characteristics.

Patient characteristic		Values (N=20)
Age (years)		
	Mean (SD)	66 (15)
	Range	27-80
Sex (assigned at birth), n (%)		
	Male	11 (55)
	Female	9 (45)
Race, n (%)		
	African American or Black	0 (0)
	Asian	3 (15)
	White	14 (70)
	Other races	3 (15)
Hispanic, n (%)		3 (15)
Education, n (%)		
	Some college	2 (10)
	College degree	11 (55)
	Advanced graduate degree	7 (35)
Household income (US $), n (%)		
	<$50,000	3 (15)
	$50,001-$100,000	6 (30)
	$100,001-$200,000	3 (15)
	>$200,000	6 (30)
	Prefer not to say	2 (10)
Employment status, n (%)		
	Part-time	1 (5)
	Full-time	6 (30)
	Retired	9 (45)
	On disability	1 (5)
	Unemployed	1 (5)
	Homemaker	2 (10)
Insurance status, n (%)		
	Current or former employer	11 (55)
	Direct from company	1 (5)
	Medicare	6 (30)
	Medicaid	1 (5)
	Other	1 (5)
Relationship status, n (%)		
	Never married	4 (20)
	Married	11 (55)
	Divorced	1 (5)
	Separated	1 (5)
	Widowed	3 (15)
Prior experience with virtual reality, n (%)		
	No experience	11 (55)
	A little bit of experience	5 (25)
	Some experience	2 (10)
	Quite a bit of experience	2 (10)
Hyperlipidemia, n (%)		14 (70)
Hypertension, n (%)		11 (55)
Coronary artery disease, n (%)		8 (40)
Overweight (BMI 25‐30), n (%)		8 (40)
Valvular disease or replacement, n (%)		7 (35)
Former smoker, n (%)		7 (35)
≥1 Myocardial infarction or multivessel CABG^a^, n (%)		5 (25)
History of atrial fibrillation, n (%)		5 (25)
Obesity (BMI≥30), n (%)		5 (25)
Diabetes mellitus type 2, n (%)		4 (40)
Diastolic heart failure, n (%)		3 (15)
Currently in cardiac rehabilitation, n (%)		3 (15)
Previous cardiac rehabilitation, n (%)		3 (15)
Heart transplant, n (%)		1 (5)
Anxiety, n (%)		9 (45)
Depression, n (%)		9 (45)
Chronic pain, n (%)		7 (35)
Insomnia, n (%)		4 (20)
Migraine, n (%)		3 (15)

a CABG: coronary artery bypass graft.

### Survey Outcomes

#### PSS-10, STAI-S, and ITQ

Baseline PSS-10 results showed a mean score of 19 (SD 6; range 4-32). Comparing each PSS-10 score to the sex, age, and race-related norm categories provided in the PSS-10 item inventory revealed that 18 of 20 participants had greater than average stress [31]. Pre-STAI-S scale also suggested elevated current stress with a mean of 35 (SD 12), median of 31 (IQR 28-38), with a range of 21 to 71 on a possible scale of 20‐80. In total, 1 had “no anxiety” (20-21), 14 had “low anxiety” (22-37), 2 had “moderate anxiety” (38-44), and 3 had “high anxiety” levels (45-80) [40].

The primary outcome, Δ-STAI-S scale, showed a median decrease of 7 (median 31, IQR 28-38 vs median 24, IQR 21-29; *P*<.001) and an average decrease of 8.5 (SD 9.8) or 21%. This corresponded to a large effect size (*r*=0.77). Both the median and average change were larger (ie, more negative) than the threshold of clinical significance set at −5 units. In total, 12 of 20 (60%) participants reached this threshold. A total of 1 of 20 (5%) participants had an increase equal to the threshold (ie,+5 units).

The mean for the cohort on the ITQ was 59 (SD 12.6) with a range of 47 to 90 on a maximum scale of 18 to 126. The majority of participants reported negligible (n=11) or minimal (n=1) “cybersickness,” while 5 had “significant” symptoms, and 3 fell into the “bad” range [53]. Of note, those who did report symptoms rated each as mild or moderate. None were rated as severe. Fatigue was the most commonly reported symptom. The mean rating of the VR experience overall was 9 of 10 (SD 2; median 9, IQR 8-10; mode 10) with one outlier who gave a rating of 3. In total, 11 individuals had no previous experience with VR, 2 had a little bit, 1 had some, and 2 had quite a bit.

Finally, multiple regression analysis was conducted to examine the relationship between the variable: Δ-STAI-S scale and various potential predictors gathered through the surveys including STAI-S pre-experience score, PSS score, SSQ score, ITQ score, overall VR rating, age, and sex. The initial model demonstrated good fit and accounted for 86% of the variability in Δ-STAI-S (multiple *R*=0.96; adjusted *R*^2^=0.86; ANOVA of regression: *P*<.001). However, only the STAI-S scale pre-experience score (*P*<.001) and overall rating (*P*=.04) were significantly correlated with Δ-STAI-S. After excluding the nonsignificant variables, the model showed that both variables had negative coefficients that were significantly correlated with Δ-STAI-S, indicating that those with higher scores on these variables tended to have a greater decrease in STAI-S scale. The model with the final 2 predictors produced multiple *R*=0.95, adjusted *R*^2^=0.90, ANOVA of regression: *P*<.001 (Multimedia Appendix 1).

#### Physiologic Outcomes: BP, HR, and GSR

**Figure 3.**
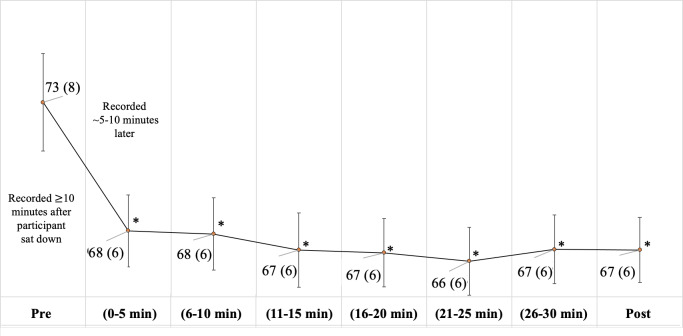
Change in average heart rate (beats per minute). Error bars represent SE. Post: recorded 5 minutes after experience; Pre: recorded 5-10 minutes before experience. *P<.001.

There was no significant change in systolic or diastolic BP among participants (Table 2). Pre- versus postexperience HR recordings demonstrated a statistically significant decreased mean HR of nearly 6 beats per minute. Upon examination of longitudinal models with spaghetti plots, it was observed that this drop tended to occur between time point 1 (pre-experience) and time point 2 (0 to 5 minutes), followed by a plateau (Figure 3). Note that findings remained significant when adjusted for multiple statistical comparisons using the Bonferroni correction as well as Holm and Hochberg tests. The change in GSR conductance and area under the curve (before VR vs after VR) did not demonstrate a significant change (Table 2). GSR was only analyzed before and after the VR experience because of technical issues that affected data collection for 5 of 20 participants.

**Table 2.** Change in State Trait Anxiety Inventory-State (STAI-S) scale, blood pressure (BP), and galvanic skin response (GSR) (N=20).

Variable	Pre-VR^a^		Post-VR		Δ (post-pre)^b^		95% CI	*P* value
	Mean (SD)	Median (IQR)	Mean (SD)	Median (IQR)	Mean (SD)	Median (IQR)		
STAI-S (points)	35 (12)	31 (28-38)	26 (6)	24 (21-29)	−8.5 (9.8)	−7 (−11.5 to −3)	−11.5 to −4	.001^c^
Systolic BP (mm Hg)	128 (14)	127 (122-133)	129 (18)	128 (115-138)	1 (13.8)	1 (−9 to 6.5)	−5.5 to 7.5	.75
Diastolic BP (mm Hg)	79 (9)	78 (71-85)	80 (10)	79 (75-89)	1 (7)	1 (−3 to 4)	−2.3 to 4.2	.60
Heart rate (beats per minute)	73 (8)	72 (67-78)	67 (6)	65 (63-70)	−6 (5)	−7 (−8 to −3)	−8.4 to −3.5	<.001
GSR (microsiemens)	0.74 (0.89)	0.48 (0.28-0.79)	0.70 (0.57)	0.61 (0.32-0.82)	−0.036 (0.42)	0.11 (−0.17 to −0.3)	−0.2 to 0.2	.50^c^
GSR AUC^d^ (microsiemens*10^−1^ second)	1979 (2518)	1308 (836-2162)	1933 (1701)	1726 (854-2191)	−46 (1183)	317 (−307 to 504)	−256 to 448	.41^c^

a VR: virtual reality.

b Δ: change or difference.

c Indicates Wilcoxon signed rank sum test (otherwise 2-sample paired *t* test used).

d AUC: area under the curve.

#### Physiologic Outcomes: Time-Domain HRV Parameters

**Figure 4.**
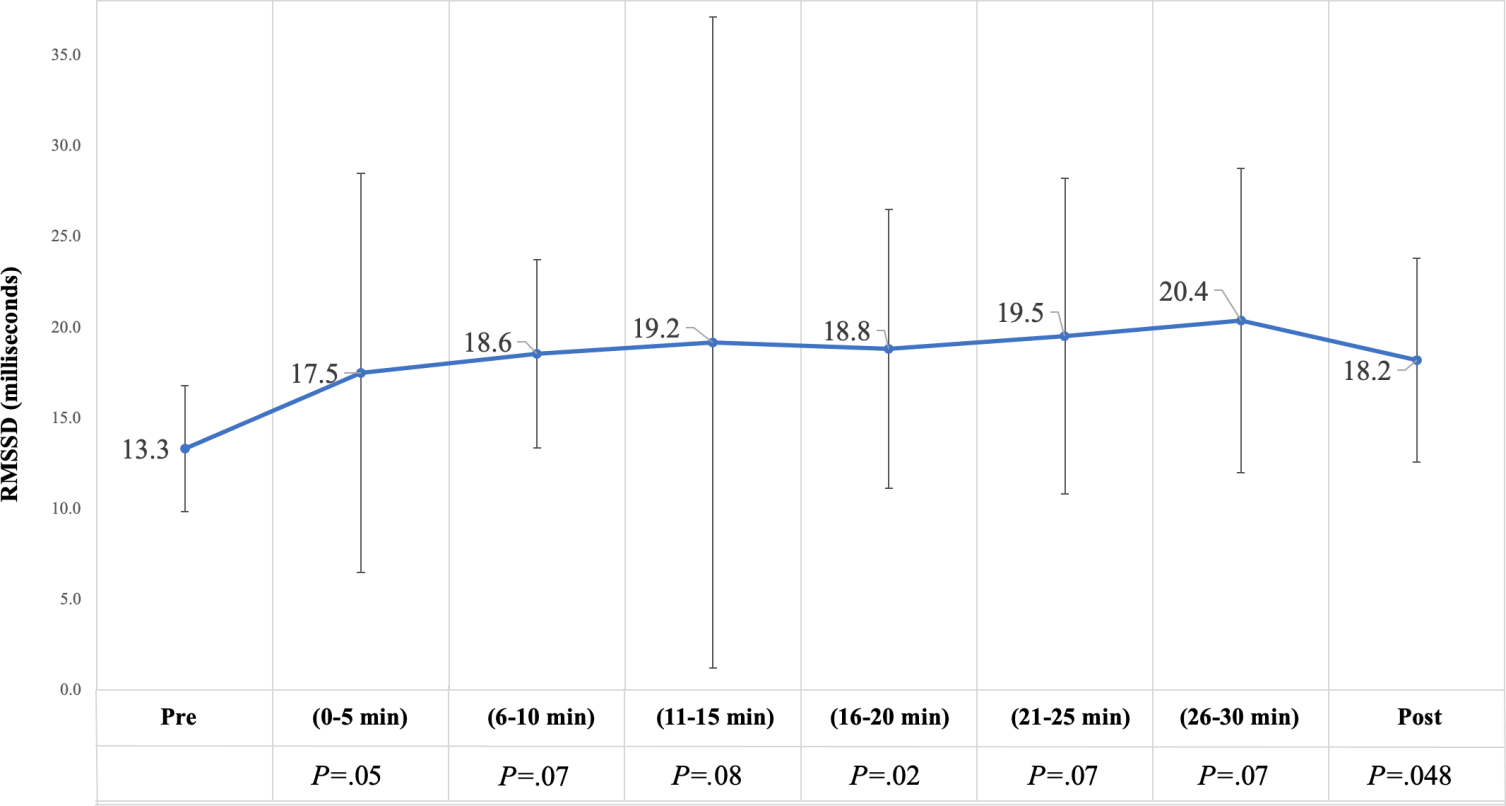
Change in median RMSSD (milliseconds). Error bars represent SE. Post: recorded 5 minutes after experience; Pre: recorded 5-10 minutes before experience; RMSSD: root-mean-square of successive differences.

The median RMSSD (milliseconds) for the cohort was increased at all time points relative to baseline (highest at time point 26‐30 minutes) but was significantly increased from baseline only at 16‐20 minutes and after the experience (Figure 4). Median SDNN (milliseconds) also tended to increase across time, though the change from baseline was only statistically significant after the experience (Multimedia Appendix 1).

#### Physiologic Outcomes: Frequency-Domain HRV Parameters

**Figure 5.**
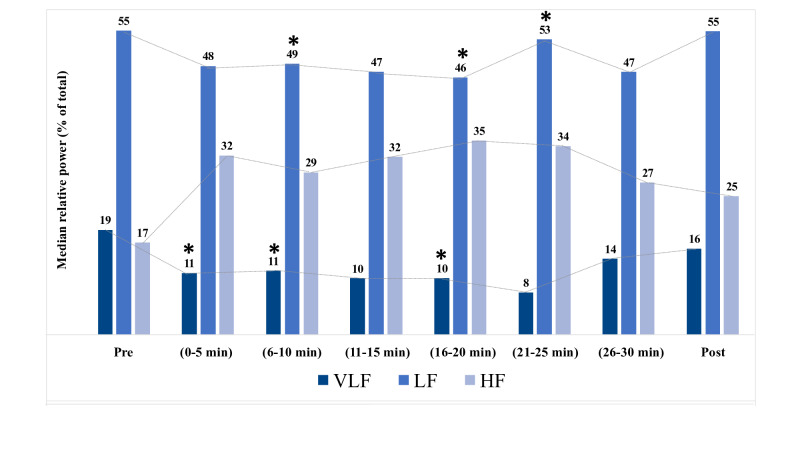
Change in relative frequency band power. HF: high frequency; LF: low frequency; Post: recorded 5 minutes after experience; Pre: recorded 5-10 minutes before experience; VLF: very low frequency. *P<.05 (see Multimedia Appendix 1 for specific values).

**Figure 6.**
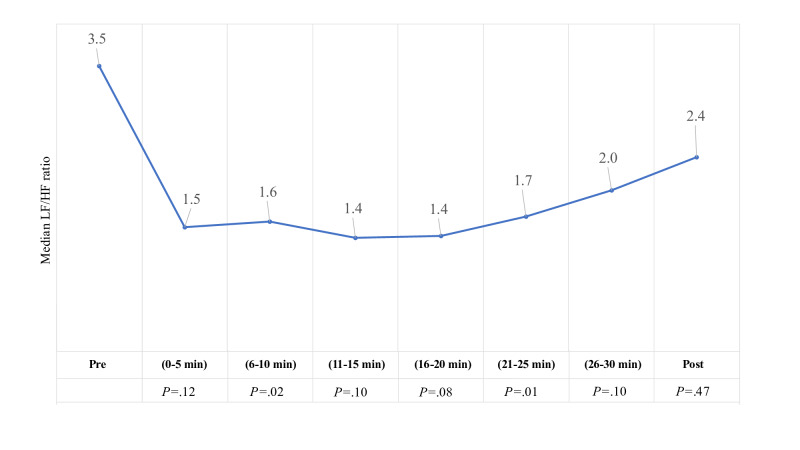
Change in median LF:HF ratio. HF: high frequency; LF: low frequency; Post: recorded 5 minutes after experience; Pre: recorded 5-10 minutes before experience.

In terms of relative power, there was a significant decrease in very low frequency power (relative to pre-experience or baseline) at 3 time points, associated with a reciprocal increase in HF, also significant at 3 time points (Figure 5). The LF:HF ratio was decreased relative to baseline at all time points, reaching a nadir in the middle of the experience and gradually increasing again during the last 3 time points (Figure 6). All median values were greater than 1, indicating that at least half of the participants had greater power from LF than HF. Note that none of the HRV findings were still significant after adjusting for multiple statistical comparisons using the Bonferroni correction and Holm and Hochberg tests.

### Qualitative Results

#### Overview

**Figure 7.**
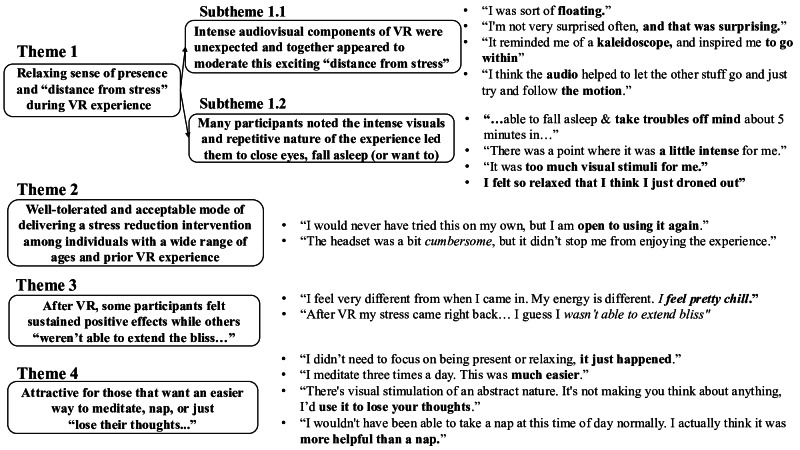
Qualitative interview results using inductive thematic analysis: 4 themes and 2 subthemes. VR: virtual reality.

In total, 6 potential themes were constructed. Upon reviewing and defining the themes, 2 themes did not have sufficient evidence to constitute substantive themes and rather reflected subthemes of theme 1. In the interviews, participants described their stress and anxiety as external and out of their control rather than describing an internal locus of control. For example, some described worry about family members, painful divorce, or daily stresses piling on from career, traffic, or their roof leaking. Others expressed stress more directly related to their cardiac conditions or adverse cardiovascular events. Figure 7 summarizes the main themes and subthemes that emerged regarding the VR experience.

#### Theme 1

During VR, participants experienced a relaxing sense of presence and “distance from stress.” Participants generally reported losing track of all sense of time or time passing more quickly while they had a sense of “sort of floating.” Others completely forgot about or felt removed from usual stress, with one person stating, “I wasn’t able to focus on other thoughts really” and another said, “I could lose myself in the patterns and everything and kind of just ... I don’t know, this is something different.” Qualitative analysis also provided insight that the intense audiovisual components of VR were unexpected and appeared to moderate this “distance from stress” (subtheme 1.1). Participants described that the combination of audio and visuals synergistically created an immersive experience that created an almost “indescribable factor.” Many participants organically offered that they were pleasantly surprised by what they experienced. Some mentioned that the red and yellow colors drew them in, while others focused on the starry sky transporting them back to being a child star gazing. Others found that the sacred geometry reminded them of a “kaleidoscope” inspiring them to “go within” or that the “motion around the geometry with the concentric circles [paired with] the audio helped to let the other stuff go.” Many participants were most surprised by the nonverbal audio, with one person noting, “I wouldn’t have listened to that on my own, but surprisingly I liked it.” Some described that they would have expected to experience something different. For one person, these intense visuals were “off-putting” with “too much going on,” and envisioning the experience would have something more relaxing like “someone walking slowly on a path.”

The next subtheme (1.2) was the intense visual experience that led participants to close their eyes (or at least want to)*.* On one end of the spectrum, some participants felt so relaxed that they felt as if they fell asleep after about 5 minutes. On the other end of the spectrum was the patient mentioned earlier who said it was “difficult to keep my eyes open” due to eye strain. Another noted, “there was a point where it was a little intense for me.”

#### Theme 2

The VR experience was well-tolerated overall and was an acceptable delivery mode for this stress reduction intervention across a wide range of ages. A few participants noted that the headset was slightly cumbersome or that their head felt slightly turned or not centered, “I loved the experience, it was very relaxing, it would have been perfect had there been a better fit with the headset/headphones.” However, most participants stated that the headset was not uncomfortable and did not hinder their enjoyment of the content. There were few other physical sensations discussed during the interview aside from one participant describing a transient moment of “stomach dropping” and one participant who experienced eye strain. Universally, participants were open to trying VR again, including the one with eye strain (though with different content).

#### Theme 3

After taking off the headset, some participants had sustained positive effects, while others were “not able to extend the bliss.” On one end of the spectrum, one participant noted that his busy mind and daily stresses came flooding back to him when he took off the headset and especially once he was handed the postexperience survey. Another noted, “I mean, I do feel relaxed. I do feel present. I do feel energized and clear, much more clear than when I came in the room. But now there’s also that old [stress] too, of like yeah, I remember that.” Others had a change in mood and energy that was visible and palpable to the interviewer, with one participant feeling more confident, “I just feel more sure of myself. And I’m able to push all my worries about my daughter, more away.” One person reflected, “my energy is different now ... under stressful times, you’re thinking about [many things], but then when I did this, I was just like ... now I feel pretty chill.” While poststudy visit follow-up was not part of the protocol, one participant reached out the next day just to the study team to say that they had “felt a sense of calm and peace that last well into the evening ...”

#### Theme 4

Participants felt that this VR experience may be helpful for those who want an easier way to meditate, nap, or just “lose their thoughts” for a bit. Multiple participants referred to the “abstract nature of the visual stimulation” in particular or losing themselves in the patterns. A few participants compared the VR experience to other stress reduction methods, such as meditation, progressive muscle relaxation, or even taking a nap. They proposed that it could be used “as an interlude for daily stress” and would recommend it to others as “a good tool to mellow out in the moment.” While the state of mind and feelings seemed to be described as similar to those induced by these techniques, they described that “[VR] was much easier” and for one they thought it could even “replace my regular meditation practice.” This final theme also underscores the participants’ experience of this VR intervention and future therapeutic value.

### Mixed Methods Analysis

The VR intervention by mixed methods analysis had a positive effect on stress reduction based on (1) the statistically (median STAI-S Δ=–7*u*; *P*<.001) and clinically significant (minimal clinically important difference=STAI-S scale Δ5*u* or one half of a SD for this health-related quality of life scale) [59] decrease in STAI-S scale scores, (2) the statistically significant decrease in HR (−6 beats per minute; *P*<.001), and (3) qualitative interview feedback describing that the intervention induced a relaxing sense of “distance from stress” that was associated with a change in energy and feelings similar to meditation or a flow state. However, the remaining physiologic outcomes BP, GSR, and HRV were not significantly changed, and thus moderated the magnitude of this positive effect. Overall, survey data, physiologic data, and qualitative interviews support the conclusion that the VR intervention is safe and tolerable. Interview feedback provided further depth, with most participants describing an enjoyable experience that they were open to doing again.

## Discussion

### Overview

Our study found that a sample of patients with CVD or risk of CVD had above-average stress. A statistically and clinically significant decrease in subjective perception of stress was observed after participants experienced a novel VR application. HR significantly decreased, while BP and GSR remained the same. Changes in HRV parameters were consistent with increased vagal tone over time but were only statistically significant at certain time points (before multiple comparisons adjustment). Overall, the VR intervention was a safe and feasible stress reduction method.

### Principal Findings

Our pilot study of a VR experience for patients with or at risk for CVD demonstrated several interesting findings. First, this sample of patients had higher than average stress based on preintervention stress scores, with nearly half having EMR evidence of anxiety or depression, which is consistent with previous epidemiological data of patients with coronary artery disease and those who have experienced severe cardiac events [1,3-7]. This finding also suggests a need to address mental stress in patients with or at risk for CVD. The STAI-S scale is widely validated with high internal consistency (0.86‐0.95) and reliability (0.65‐0.75) coefficients [40], while PSS-10 was also chosen for its validity and reliability [32]. Higher PSS-10 scores have been associated with failure to quit smoking, worse control of blood sugar, greater vulnerability to depressive symptoms, and more frequently having the common cold [31]. Qualitative analysis provided additional scope to the finding of elevated average stress in our cohort (Figure 7).

Second, there was a statistically (median Δ=–7*u*; *P*<.001) and clinically significant (minimal clinically important difference=Δ5*u* or one half of a SD for this health-related quality of life scale) [59] decrease in subjective perception of stress measured by survey data that matched theme 1 and was overall supported by qualitative feedback. Previous research suggests that VR exerts its effect by temporarily silencing the DMN and can lead to the state of mind opposite to that produced by the DMN, coined “flow” [64]. Often described as “being in the zone,” the 4 attributes of flow include selflessness, timelessness, effortlessness, and richness [65]. It is thought that flow requires “transient hypofrontality” or a temporarily suppressed prefrontal cortex that allows the self-critical mind to quiet and connect neural areas that do not normally communicate [66]. This “lateral thinking” [18] can create the opportunity for new insights and a profound sense of calm.

VR experts propose the following mechanism by which VR can induce flow within minutes: VR is completely immersive, so that it can capture the user’s full attention like no other audiovisual medium. By removing distractions and offering a novel, information-rich experience, it can interrupt the noisy DMN, change brainwaves from β to α, and increase neurohormonal signaling through dopamine, oxytocin, and serotonin that has the effect of creating an aura of tranquility that can conjure flow [18]. VR was shown to produce a nearly identical state of consciousness as psilocybin, a known flow-inducing compound, using a VR program called the Hallucination Machine that radically altered visual perceptions [20]. While psychedelics have been an area of booming interest for the treatment of severe anxiety, phobias, depression, autism, and posttraumatic stress disorder, these findings demonstrate exactly why VR continues to expand in the area of psychiatry and can be applied generally to stress reduction.

The physiologic data did not uniformly converge with these subjective findings. On the one hand, HR significantly decreased, and HRV parameters showed significant changes consistent with increased parasympathetic state at certain time points. Pre- versus post-HR recordings demonstrated a statistically significant decreased mean HR of nearly 6 beats per minute. Longitudinal plots showed that the initial drop tended to occur between the first 2 time points and then persisted. The protocol attempted to allow time for participants to reach resting HR before the first recording (completing consent, pre-experience survey, and connecting chest strap and GSR sensors while in a seated position). For most healthy adults, HR stabilizes after 4 minutes of inactivity [67]. So, it is possible that this represents a true effect related to VR. However, if our sample of participants required longer for cardiovascular activity to return to the resting state due to their health state or there were other factors that interfered (such as mental stress provoked by completing the presurvey), then it is plausible that the observed results are due to the passage of time. This cannot be deciphered without having a placebo-controlled trial.

RMSSD (mainstream apps such as Elite HRV and Whoop report this as an “HRV score,” with higher scores generally indicating better “readiness” for the body to adapt and perform), SDNN, relative power of HF band, and LF:HF ratio showed changes consistent with a more relaxed state between the first 2 time points. HRV findings were most consistent with increased vagal tone. The regulatory mechanisms at play for “short-term” HRV measurements (5-minute recordings) include changes in HR driven by changes in respiration (respiratory sinus arrhythmia), the baroreceptor reflex (negative-feedback regulation of BP), and rhythmic changes in vascular tone [56]. A period of HRV monitoring can be studied using time-domain parameters, which look at aspects of the variability in the interbeat interval, and frequency-domain parameters, which look at the distribution of power generated by each beat (by breaking the total power into 4 frequency bands) [56,57]. HRV provides a measure of how well the cardiovascular system can rapidly adjust in response to sudden physical and psychological disruptions to homeostasis. The Fast Fourier Transformation method was preferred over the autoregressive HRV analysis method because several studies have found that the autoregressive method did not produce reliable results in some patients with diabetes or hypertension [68], which we expected to be prevalent in our population.

On the other hand, BP, GSR, and at several time points, HRV parameters showed no significant change. Further, when adjusting for multiple statistical comparisons, HR changes remained significant, while HRV changes were no longer significant, indicating that these findings may be an artifact or type I error. It has been repeatedly shown that breathing exercises can cause a modest but significant decrease in HR (−2.41 beats per minute; *P*=.03) and BP (systolic: −7.06; *P*<.01 and diastolic: −3.43; *P*<.01) [69]. If there was a change in breathing pattern or vagal tone, consistent with the observed HRV trends, it could partially explain the decreased HR, but we would have also expected BP to fall. Note, however, that BP was not measured longitudinally to minimize patient disturbance. Similarly, we hypothesized that there would be a significant reduction in GSR since some studies have shown that during meditation and after consistent meditation, participants had a statistically significant reduction in GSR compared to controls [47-49]. All of these physiologic measures are subject to influence from diurnal fluctuations, positioning, recency of physical activity, caffeine intake, and sleep [56,70]. These conditions were not standardized in our study.

It is also important to note that this was a pilot study without a control; it aimed to gain initial insights into the potential effects of the VR intervention before embarking on a full-scale randomized controlled trial. The small sample size and lack of a control group limit our ability to conclude that these observed results were due to the VR intervention, specifically. There is currently a need for studies with a larger, more diverse population and control group to be able to adequately assess the effects of this VR intervention. This study can inform such studies. Although there were mixed effects in changing physiologic measures in this study, there is other evidence that VR can lower the startle response and stress hormone levels for 12 months after treatment [28] and normalize brain function confirmed through functional magnetic resonance imaging—suggesting that VR can clearly impact the mind but also the body [29].

Importantly, quantitative and qualitative analyses showed that the VR intervention was not only a tolerable and feasible stress reduction method, but it was also enjoyable across a wide range of (1) ages and (2) prior experience with VR. The SSQ produced reassuring results that are consistent with current estimates of VR cybersickness. Approximately 60%‐95% of users experience some level of cybersickness, with 5%‐15% ending their experience prematurely due to symptom severity. The prevalence and significance of cybersickness continue to fall with technical improvements in both hardware and software [71,72]. Our intervention uses slow-moving visuals that build gradually, which hopefully makes it less jarring than highly kinetic scenes. However, the 30-minute duration may contribute to the development of cybersickness. One of the most commonly reported cybersickness symptoms was fatigue. Resting in the semirecumbent position may have contributed to this. A total of 16 of 20 (80%) participants had little or no VR experience. Universally, participants were open to trying VR again, including the one with eye strain (just with different content). According to the ITQ, the cohort varied in their likelihood to become involved or engrossed in different environments and generally tended to be prone to “moderate immersion.” While this tendency supports the idea that the effectiveness of VR is moderated by a sense of presence in the experience, there was not a statistically significant relationship between ITQ and the outcome measure.

To further explore the potential moderators of the observed changes in the STAI-S scale, we performed regression analysis. Multilinear regression findings highlight that those with higher baseline acute anxiety scores tended to have larger decreases in acute anxiety. Higher overall ratings also predicted greater Δ-STAI-S scores, possibly because they valued the experience due to its anxiety-relieving effects. Per our regression model, baseline acute anxiety and overall VR rating explained 90% of the variation in STAI-S scale change. Interestingly, age, sex, chronic stress level over the past month, SSQ, and ITQ scores were not correlated with the primary outcome, nor did they have any explanatory value for variation in STAI-S scale change. Simple linear regression demonstrated weak to moderate negative correlations between age and PSS (*r*=−0.28; *P*=.23) and STAI-S scale prescores (*r*=−0.48; *P*=.03), suggesting a trend that older participants tended to have lower current and chronic stress levels. There was a moderate negative correlation between age and magnitude of decrease in STAI-S scale (*r*=−0.46; *P*=.05), suggesting that younger participants with more baseline stress experienced greater stress reduction.

### Comparison to Prior Work

To provide context for our primary outcome, Δ-STAI-S, we compared our findings (mean −9, SD 10; median −7) with previous studies using the same outcome variable to measure stress and anxiety reduction from a VR intervention (Table 3) [73-77]. While there are several differences between these studies, they provide support that the VR content under investigation may be capable of producing similar or stronger effects compared to other VR interventions. All 6 studies demonstrated statistically and clinically significant (*P<*.05; Δ≤–5 units) change from baseline, adding to the growing body of evidence for VR as a powerful stress reduction method. Of note, Baytar and Bollucuo Lu [74] incorporated hemodynamic measures of change and saw a similar initial decrease in HR of 6 beats per minute, which was statistically significant, with an additional 2 beats per minute decrease subsequently. Only 2 studies were found that examined the effect of VR on STAI scores in a group of patients with CVD; however, both used the 6-item short form of the STAI, limiting comparison with this study [78,79]. These studies, like many others in the literature, used VR for periprocedural anxiety.

**Table 3.** Comparison of this study to previous studies using virtual reality (VR) and change in State-Trait Anxiety Inventory-State Scale as an outcome for stress or anxiety reduction.

Study	Description	Change^a^	*P* value
Makaroff et al	This study	−9 (SD 10) (median change −7)	<.001^b^
Kim et al (2021) [73]	Randomized crossover design measuring anxiety of 74 healthy adults with high stress after experiencing intentionally stressful VR followed by either BF^c^ or VR relaxation	−6 (SD 10) (Stress-VR) −6 (SD 8) (Stress-BF)	VR versus BF: 0.39 VR:<.001, BF:<.001
Baytar and Bollucuo Lu (2021) [74]	40 patients undergoing septorhinoplasty with 15 minutes of preoperative 360-degree VR content with nature scenes and “meditation music”	−7 (median change −7)	<.001^b^
Niki et al (2021) [75]	10 participants in a Japanese nursing home with two 10-minute immersive VR slideshows	−9 (after first VR) −13 (after second VR)	<.001
Brown and Foronda (2020) [76]	7 patients undergoing outpatient surgeries viewing AppliedVR modules during surgery	−13	.03
Karaman and Taşdemir (2021) [77]	30 female patients using VR during fine-needle aspiration breast biopsy versus 30female patients undergoing standard protocol	−12 (experiment) −7 (control)	<.001

a SD and median were not available for several studies.

b Indicates Wilcoxon signed rank sum test, otherwise 2-tailed *t* test used.

c BF: biofeedback.

### Strengths and Limitations

This clinical investigation of a novel VR intervention designed for stress reduction in patients with CVD or at cardiovascular risk has several strengths. The pilot examined both physiologic and subjective patient data. Our inclusion of longitudinal HRV analysis helps to better explore and measure how VR may influence complex neuro-cardiac interactions that result in less stressful states. The mixed methods design and inclusion of interviews or qualitative data analysis add richness and depth to the conclusions that could be drawn from a purely quantitative or qualitative analytical approach.

Our study also has several limitations. First, this was a small sample of well-educated, predominantly White participants, which limits the generalizability of findings to other demographic populations. Second, there was no control arm in this pilot study meant to test feasibility, look for signals in clinical outcomes, and inform subsequent studies. This limitation prevents us from concluding that the observed stress reduction was due to the VR itself and not due to other confounding factors, such as the novelty of trying VR (for 11 participants). We plan to conduct future studies with a larger, more diverse population and incorporate a control group. Third, the design could not account for several factors such as time of day, caffeine intake, exercise, and medication use. This may significantly limit the measurement of physiological data and comparisons between participants [56]. While these patient-level factors were asked about in surveys (Multimedia Appendix 1), we did not explicitly instruct participants to adjust their caffeine intake or exercise prior to the study visit, and we did not adjust for these factors in our analysis. Future studies should attempt to control for these considerations. Finally, this was also a 1-time intervention lacking a longer follow-up period to fully characterize long-term effects.

### Future Directions

Our findings warrant further research in a possible VR3 study, a randomized controlled trial that compares outcomes of an intervention and a control condition. Further, there are implications for designing a future study with a more specific population, more robust or targeted intervention, as well as for determining an appropriate control arm. There are several possibilities. One option would be to focus on a younger patient population at high clinical risk. Other options would be to focus on hospitalized patients during the periprocedural time period or in the intensive care unit. Alternatively, patients in CR may stand to benefit greatly due to depression and anxiety being highly prevalent in this population [80]. Stress reduction is one of the 3 main goals of CR. This VR intervention could be used in collaboration with a cardiac psychologist to target processing anxiety or trauma after a cardiac event, given that we found the VR experience activated certain memories and feelings in these individuals. Repeated use of the VR experience could also be considered. In terms of creating a control arm, possibilities include a traditional mindfulness meditation, progressive muscle relaxation, currently available VR programs for relaxation, an audio-only experience, or even allowing participants to try to take a nap. It will also be important to discuss possible VR improvements with designers such as using a custom headstrap mounting system to better support the weight of the display, shortening the length of the experience, changing the intensity of visuals, and potentially incorporating HRV biofeedback. Equally important will be continued partnership with patients from the specified populations to tailor VR content accordingly.

### Conclusions

This group of patients with or at risk for CVD exhibited higher-than-average stress levels, aligning with epidemiological findings. The notable reduction in perceived stress aligned with some but not all physiologic changes assessed. The VR intervention appeared to be a safe and practical method for stress reduction. Future studies are necessary to explore its effectiveness to lower stress in CVD at-risk and disease populations.

## Supplementary material

10.2196/66557 Multimedia Appendix 1 Surveys, interview guide, longitudinal statistical testing results, and other survey results.

10.2196/66557 Multimedia Appendix 2 Physiologic and survey datasets generated and analyzed during this study.

## Abbreviations

BP: blood pressure
CR: cardiac rehabilitation
CVD: cardiovascular disease
DMN: default mode network
GSR: galvanic skin response
HF: high frequency
HR: heart rate
HRV: heart rate variability
ITQ: Immersive Tendencies Questionnaire
LF: low frequency
PSS-10: Perceived Stress Scale-10
RMSSD: root-mean-square of successive differences
SDNN: standard deviation of the interbeat interval of normal sinus beats
SSQ: Simulation Sickness Questionnaire
STAI-S: State-Trait Anxiety Inventory-State
UCLA: University of California Los Angeles
VR: virtual reality
