# Supplementary material for: Novel Virtual Reality Intervention for Stress Reduction Among Patients With or at Risk for Cardiovascular Disease: Mixed Methods Pilot Study
Source: JMIR Cardio. 2025 Aug 6;9:e66557. doi: 10.2196/66557 (PMC12327699; doi:10.2196/66557)
Supplement: Multimedia Appendix 1 [file cardio-v9-e66557-s001.docx]

**Introduction**

This appendix can be used by readers to supplement the description of the pre-survey, post-survey, interview guide, longitudinal testing, and survey analysis. They are included for completeness and full data access. The surveys and interview guide could serve as models for those trying to craft their own and to see the types of questions asked that generated the qualitative findings. The regression models provide the exact set of variables that were included as well as their corresponding coefficient and p-values. In sections E-G, we include the full longitudinal statistical testing results for heart rate and heart rate variability, including the parameter SDNN, which was not presented in any table or figure in the manuscript. Providing the full sets of values could be useful to future investigators on similar projects looking for points of comparison. Lastly, the additional summative survey results on stress reduction methods, exercise, caffeine, and sleep are meant to highlight the variable experiences of our participants, which we are aware could have possibly confounded our findings.

**Table of Contents**

A. Surveys

B. Semi-Structured Interview Guide

C. Multiple Regression Models for Potential Predictors of Change in STAI-S—Initial Model

D. Multiple Regression Models for Potential Predictors of Change in STAI-S—Final Model

E. Longitudinal Statistical Testing Results: Heart Rate (beats per minute)

F. Longitudinal Statistical Testing Results: Time-Domain HRV Parameters

G. Longitudinal Statistical Testing Results: Frequency-Domain HRV Parameters

H. Additional Survey Results: Participants Experience with Stress Reduction Methods

I. Additional Survey Results: Participants Experience with Exercise, Caffeine, & Sleep

1. **Surveys**


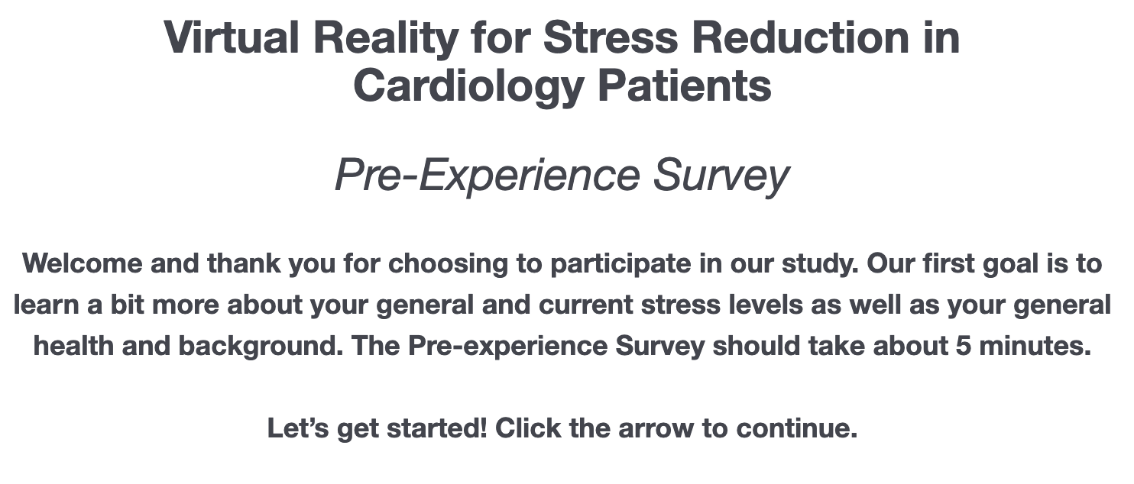


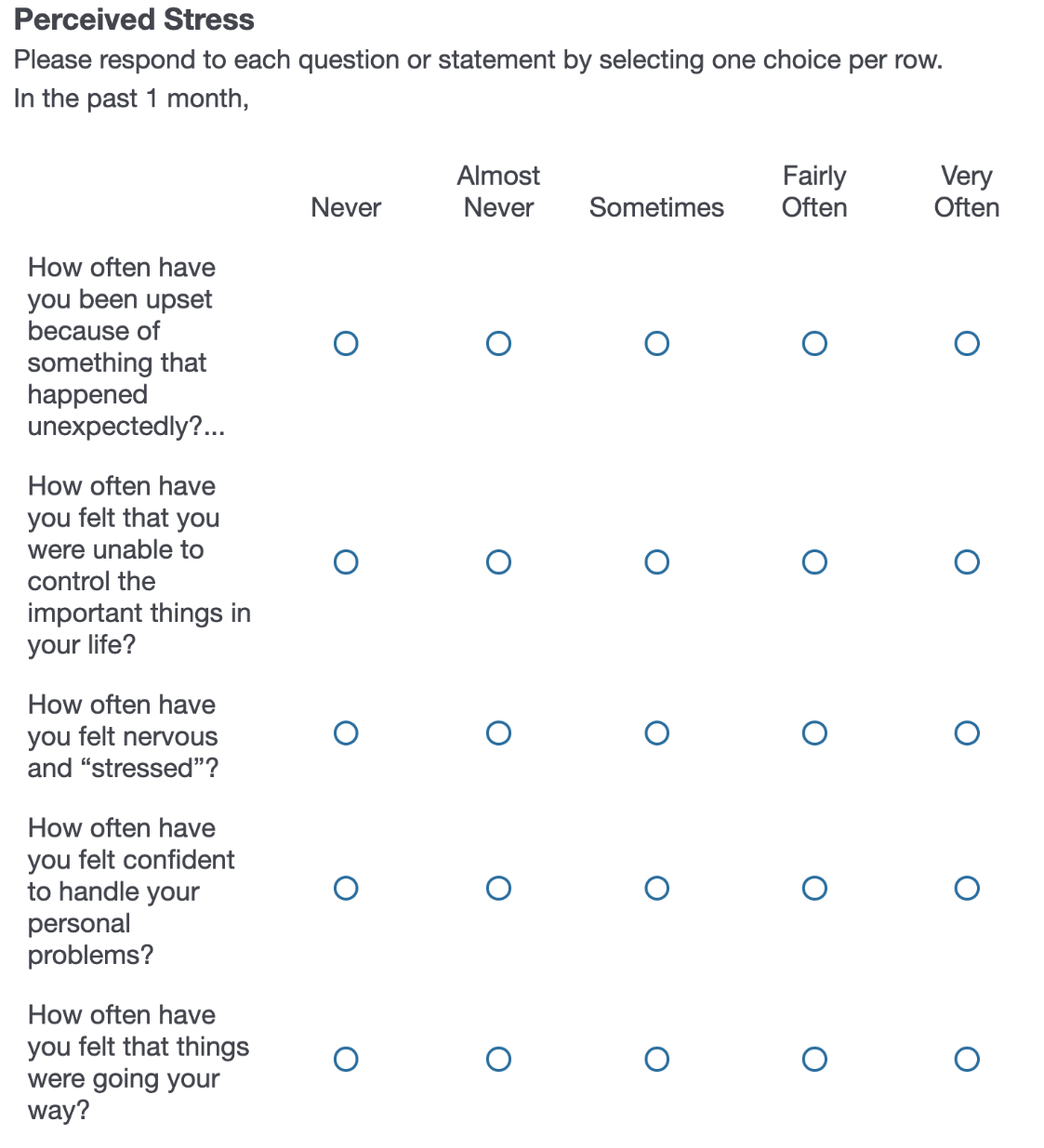


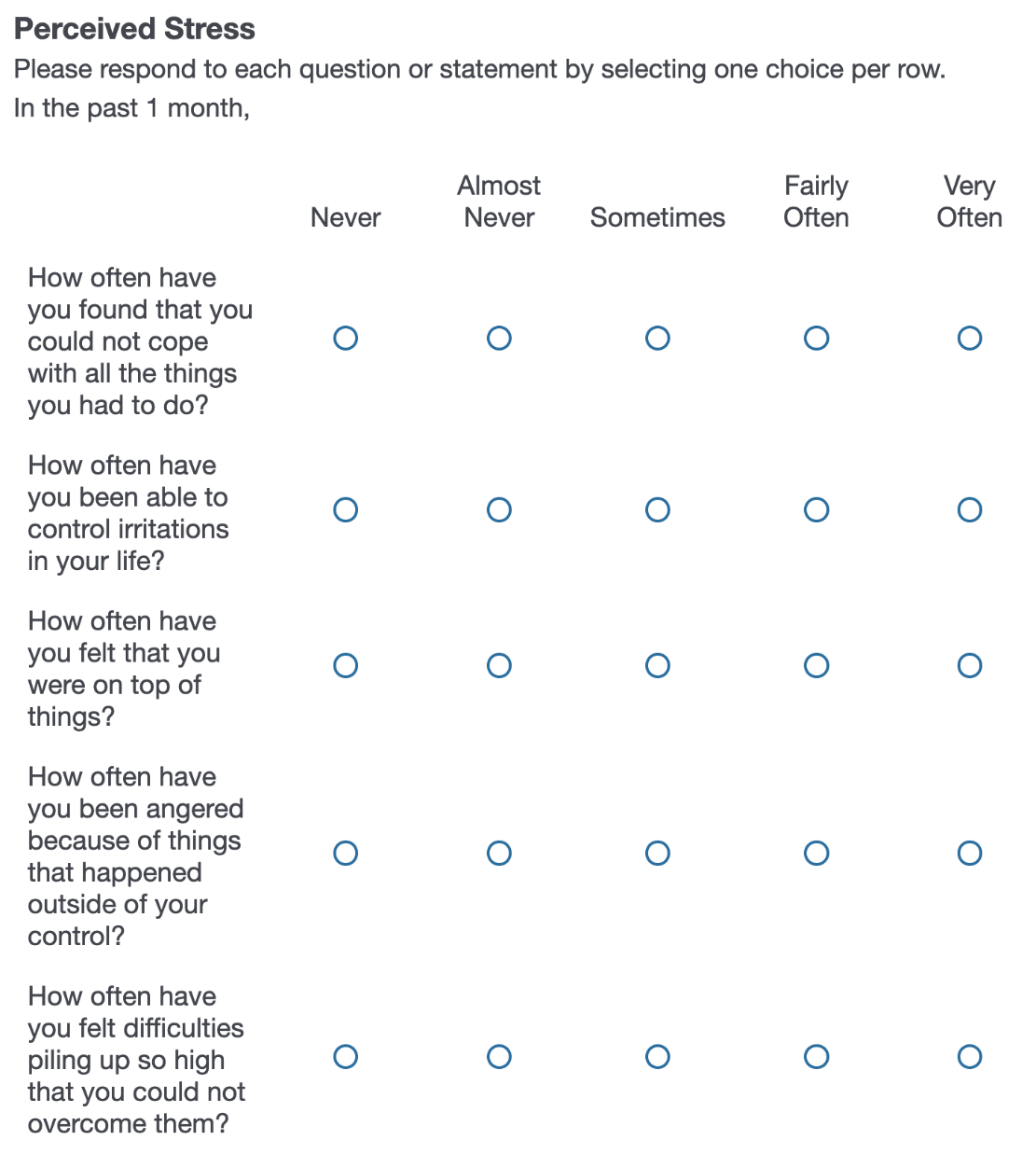


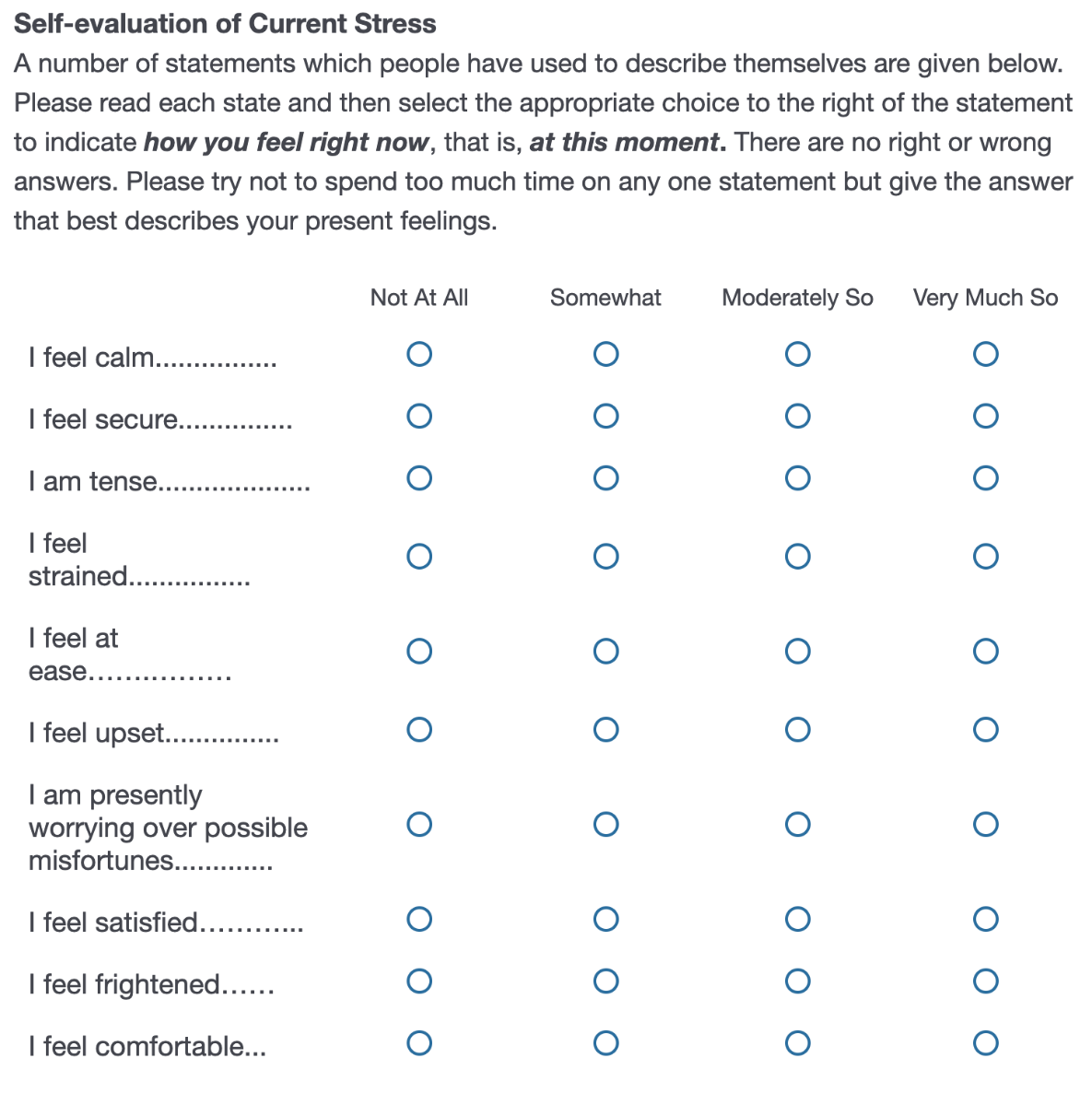


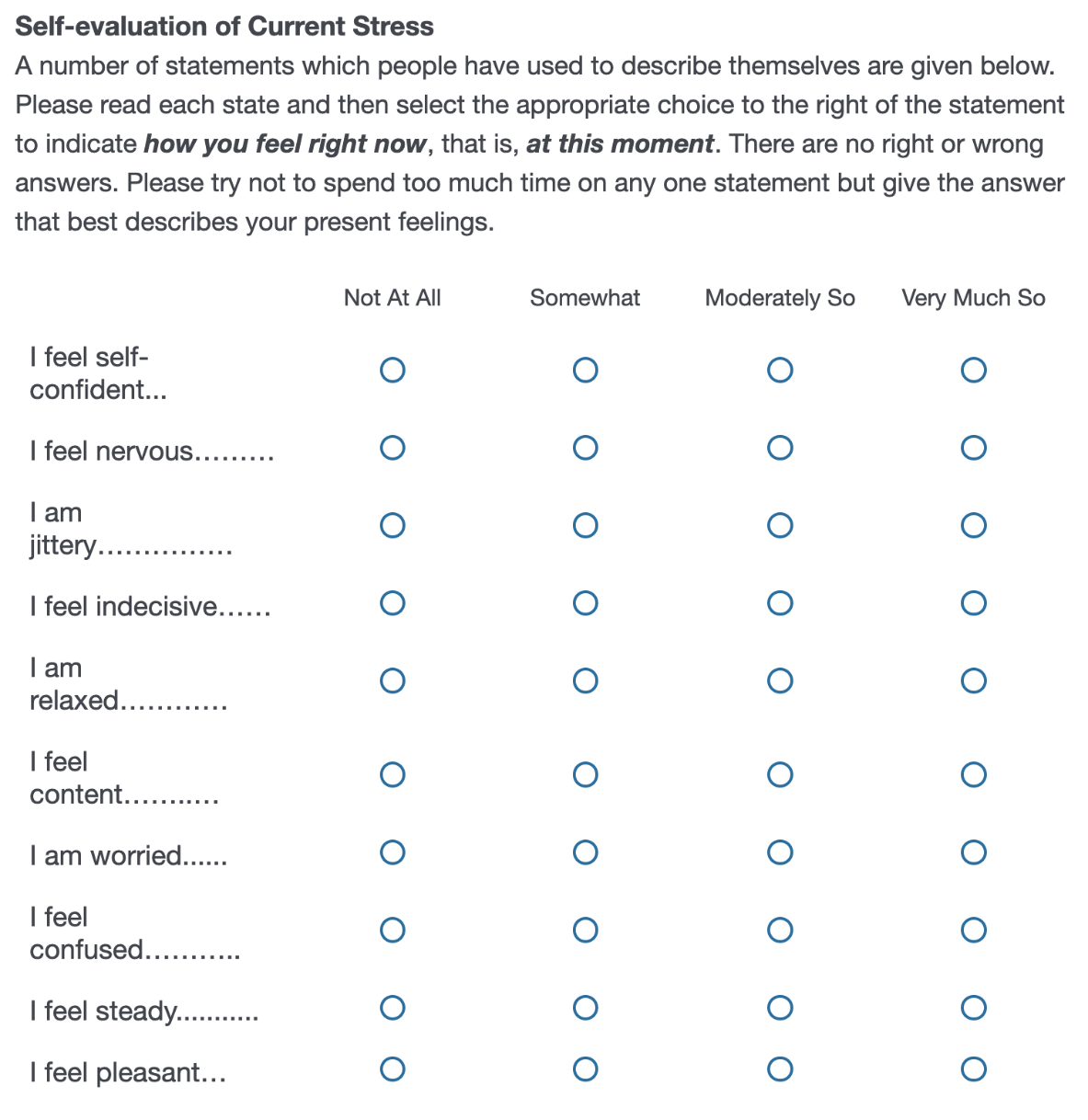


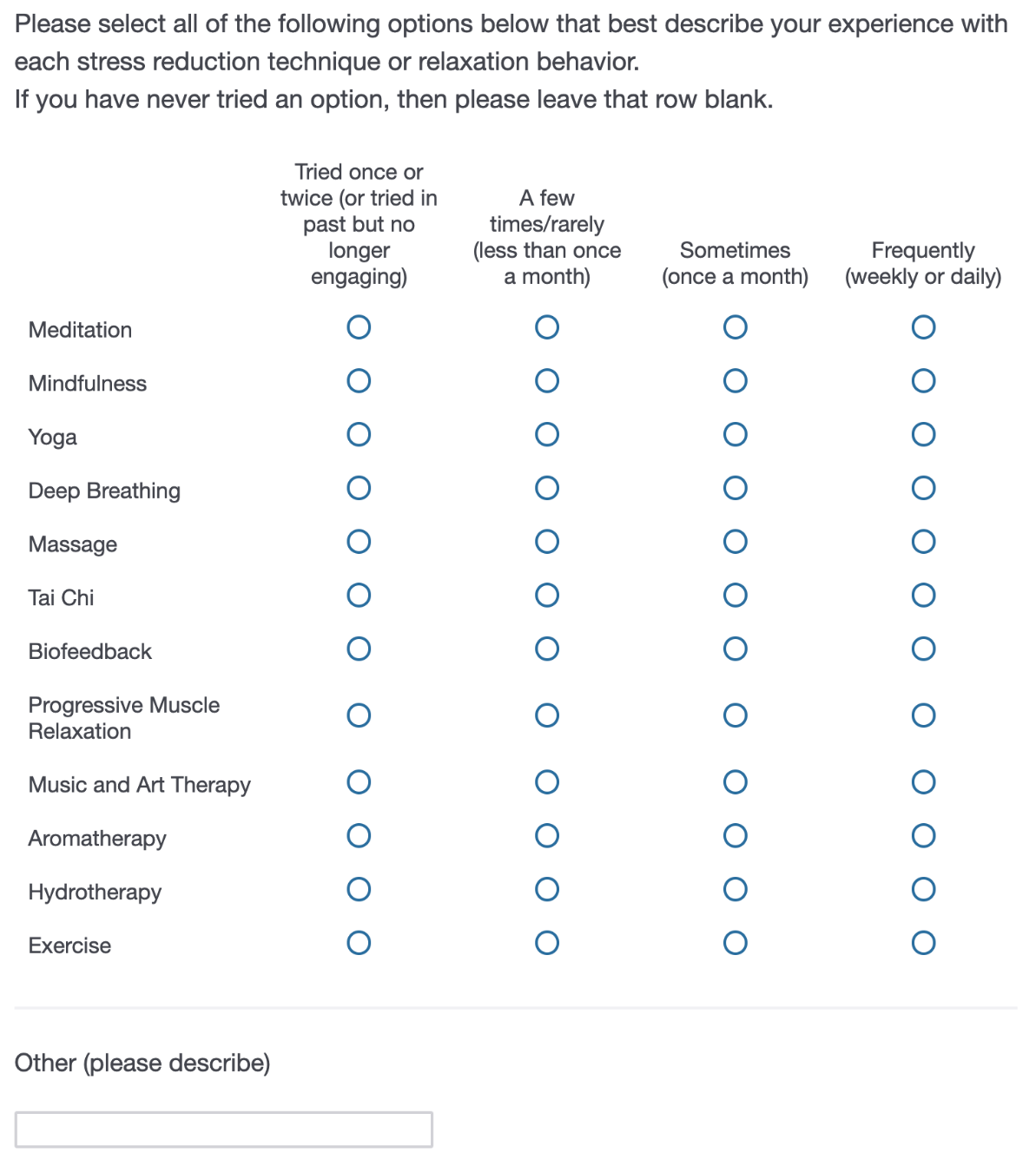


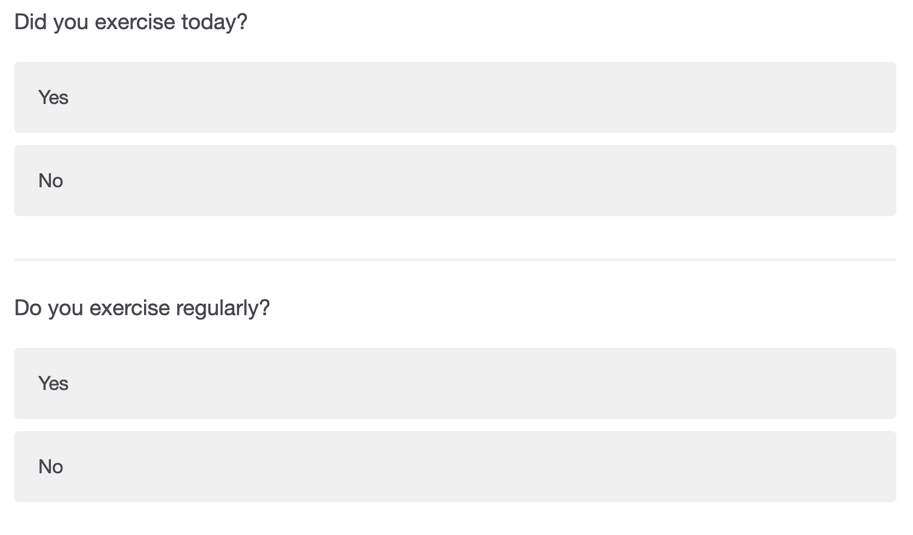


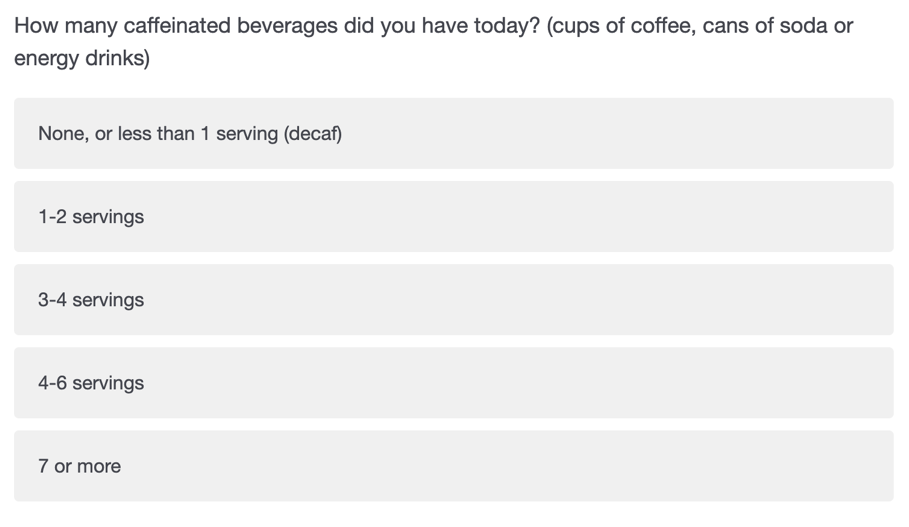


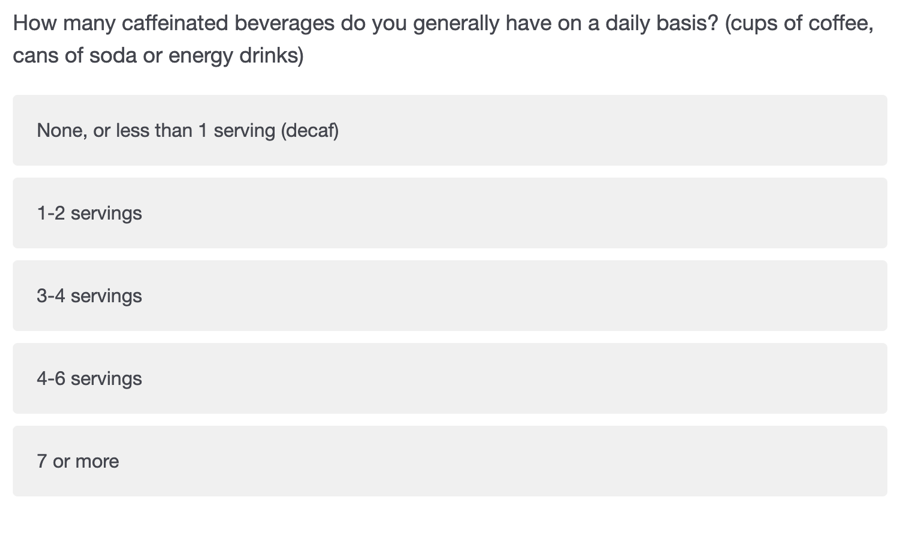


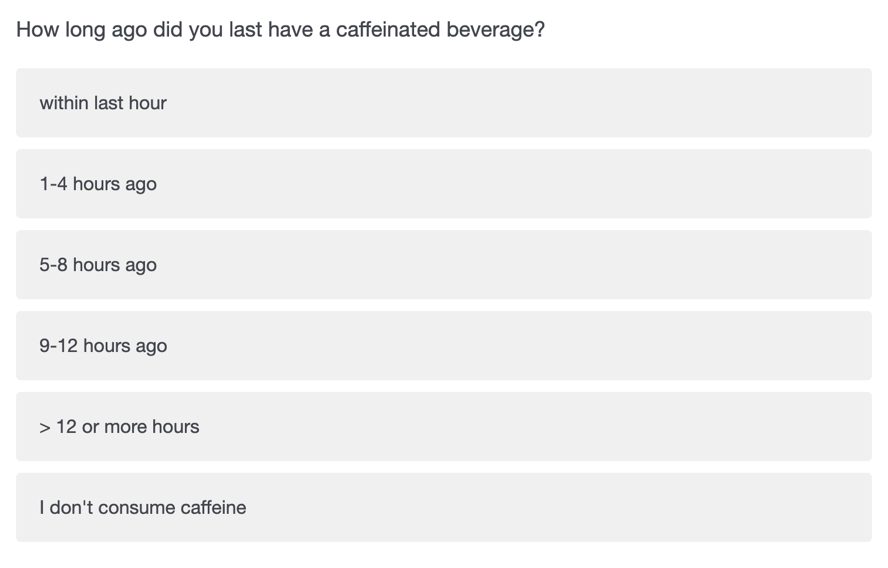


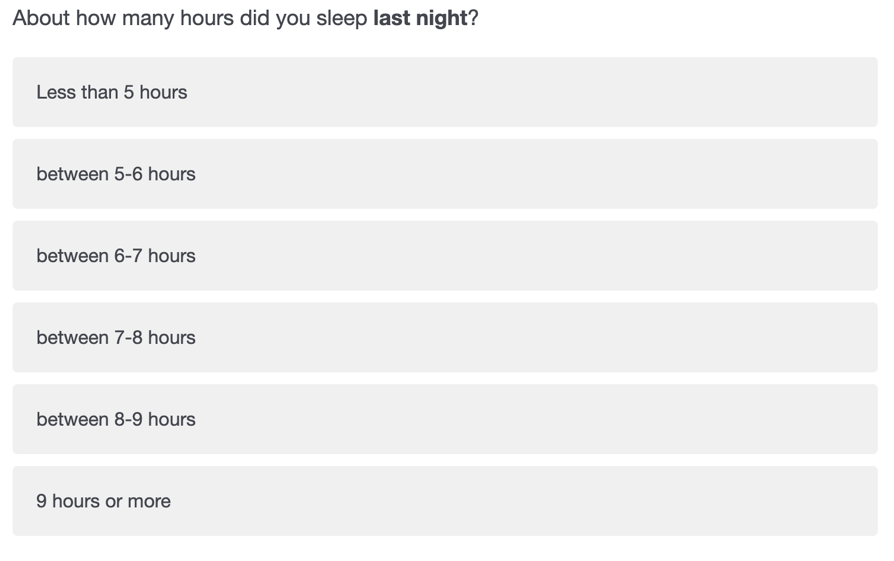


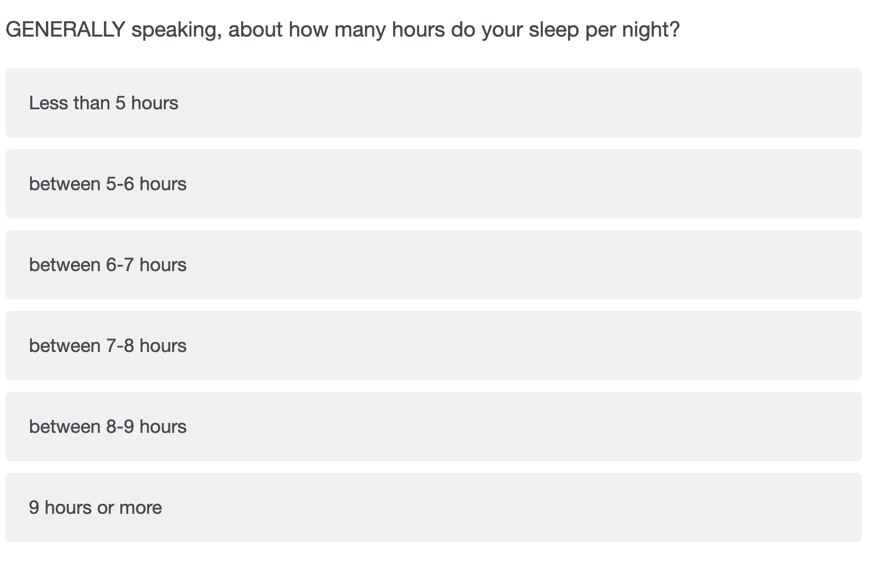

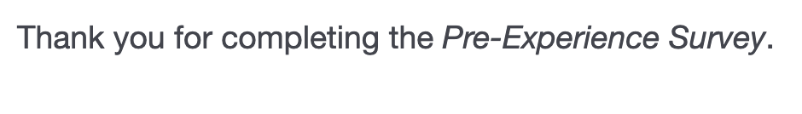


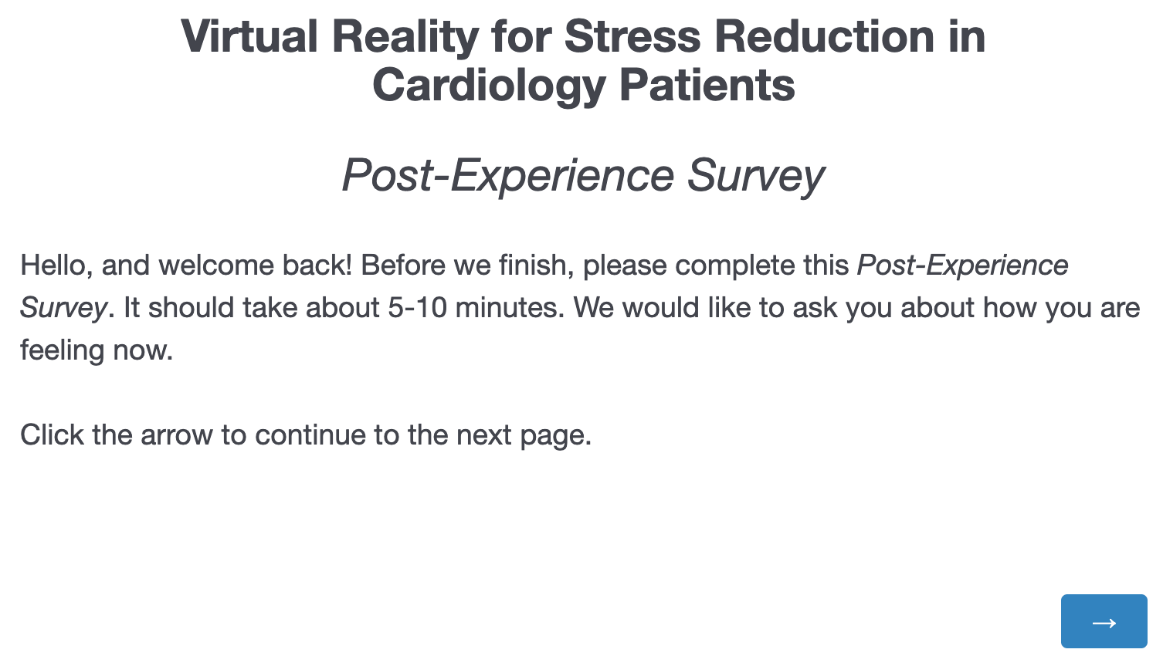


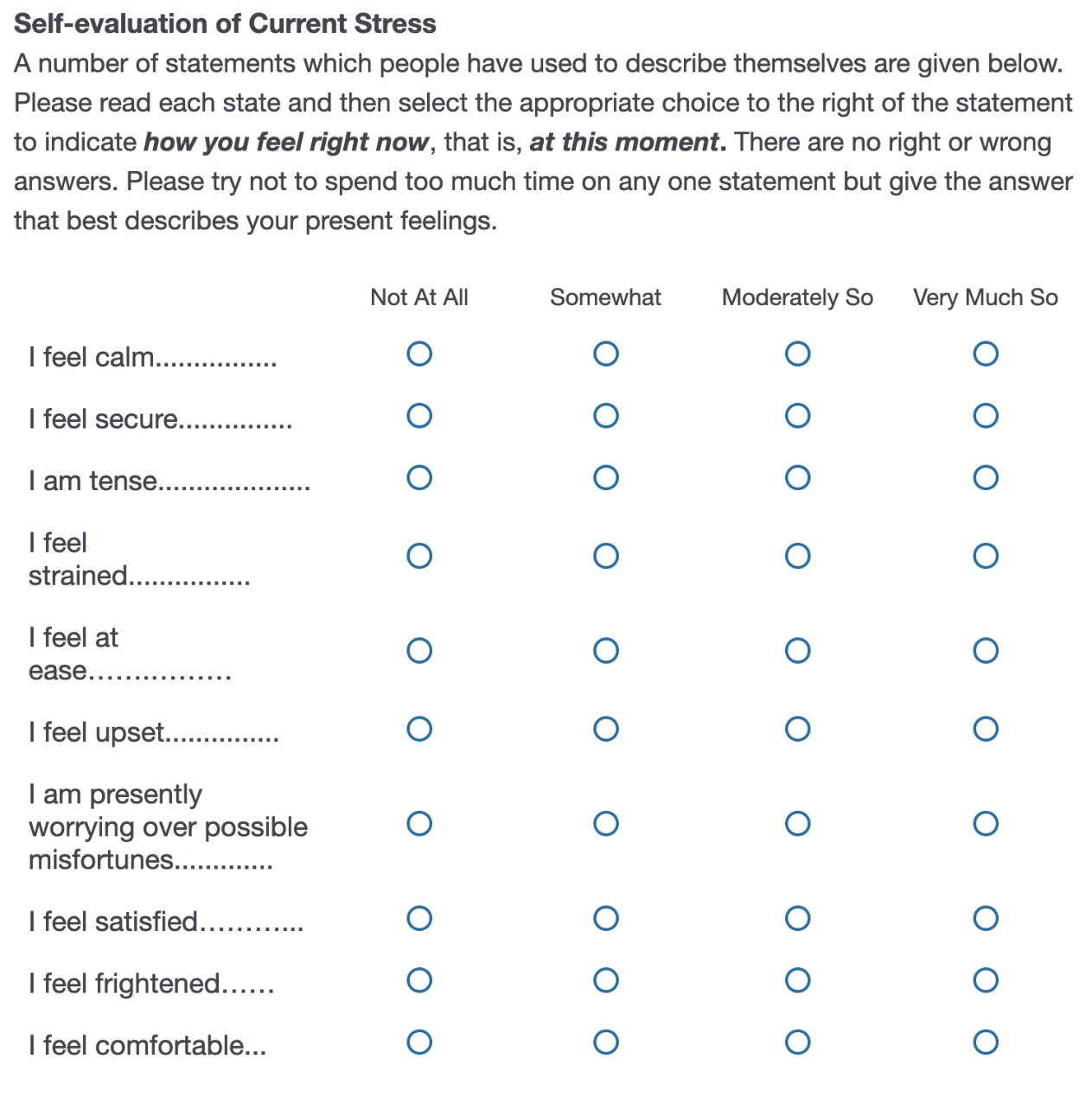


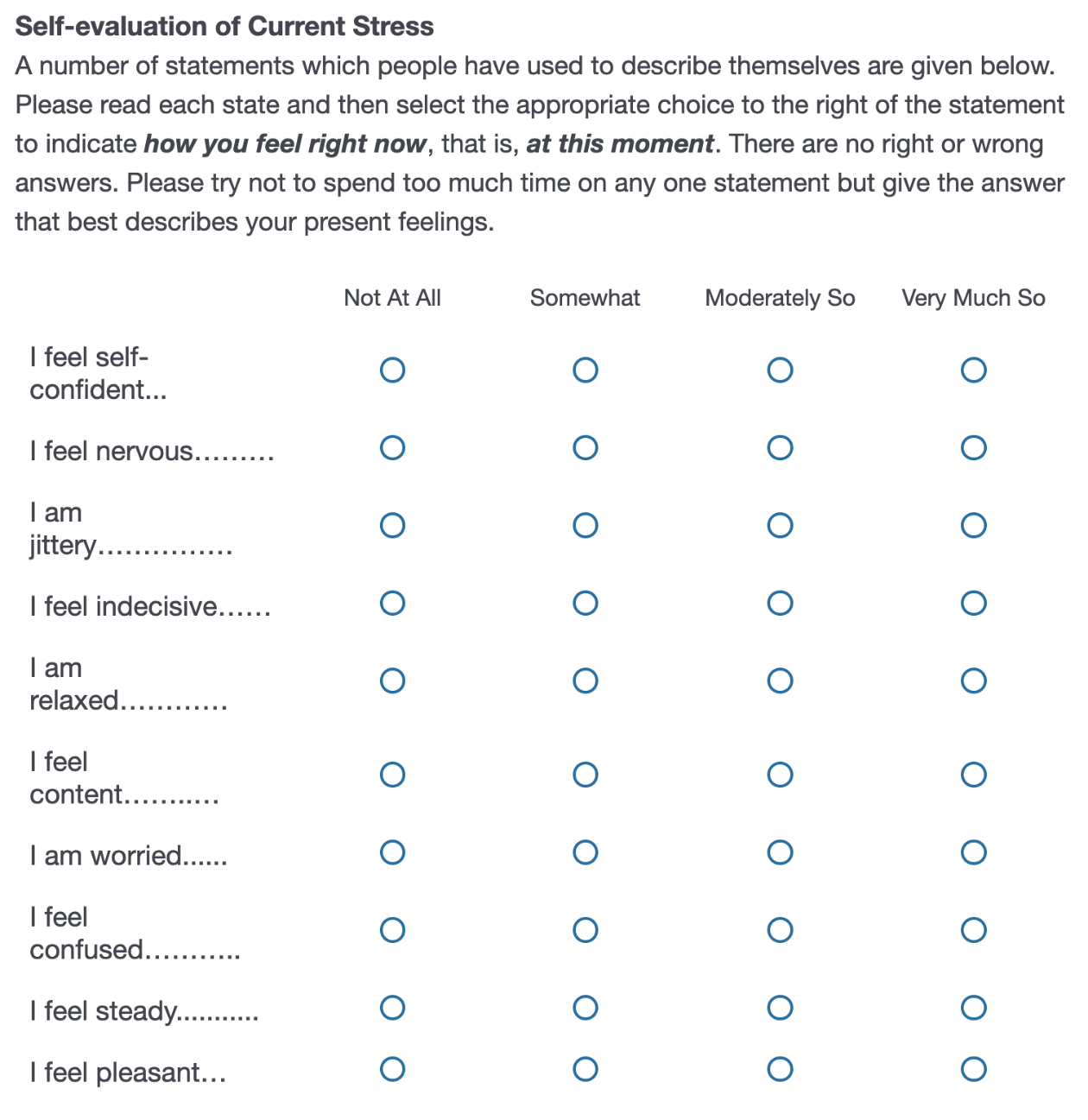


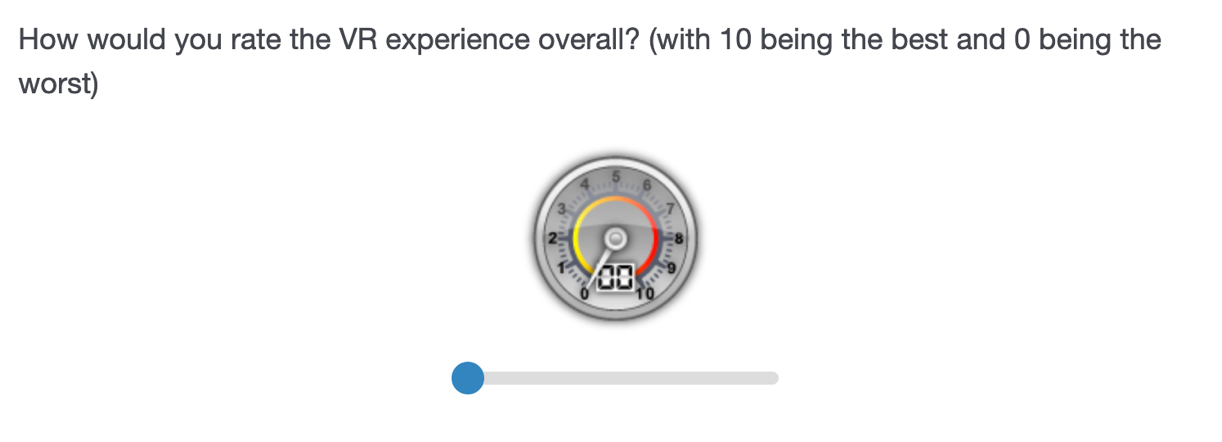


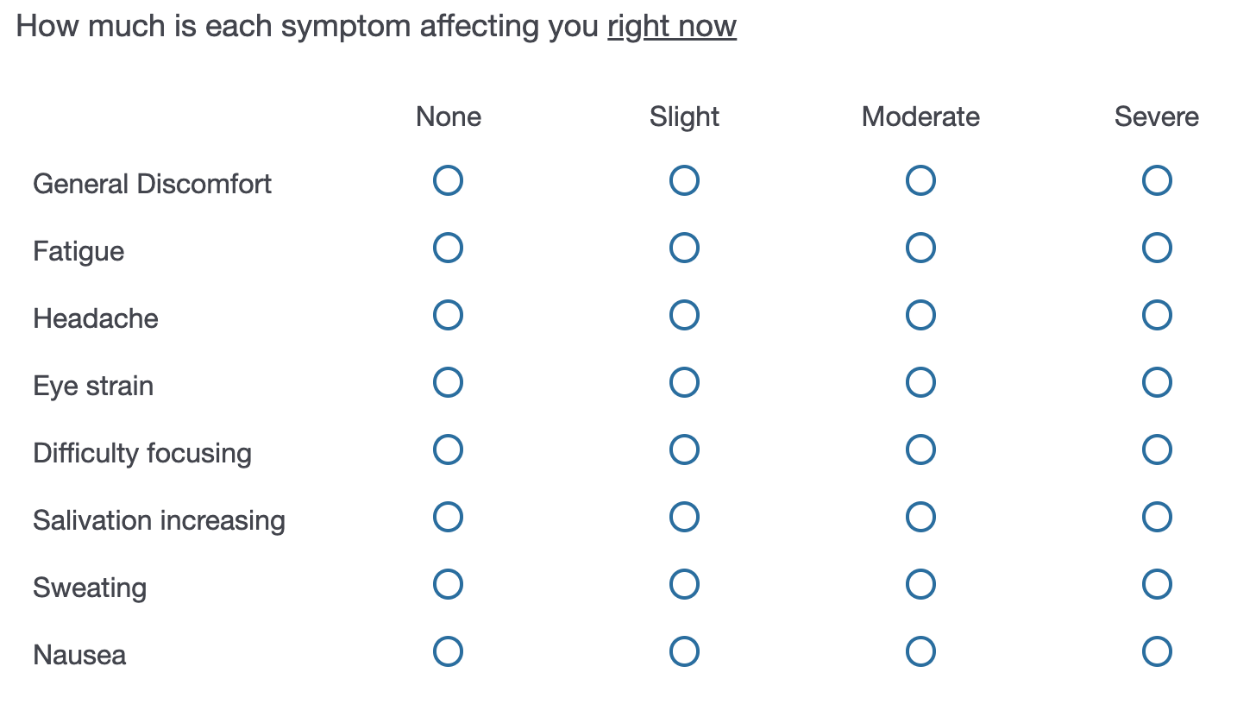


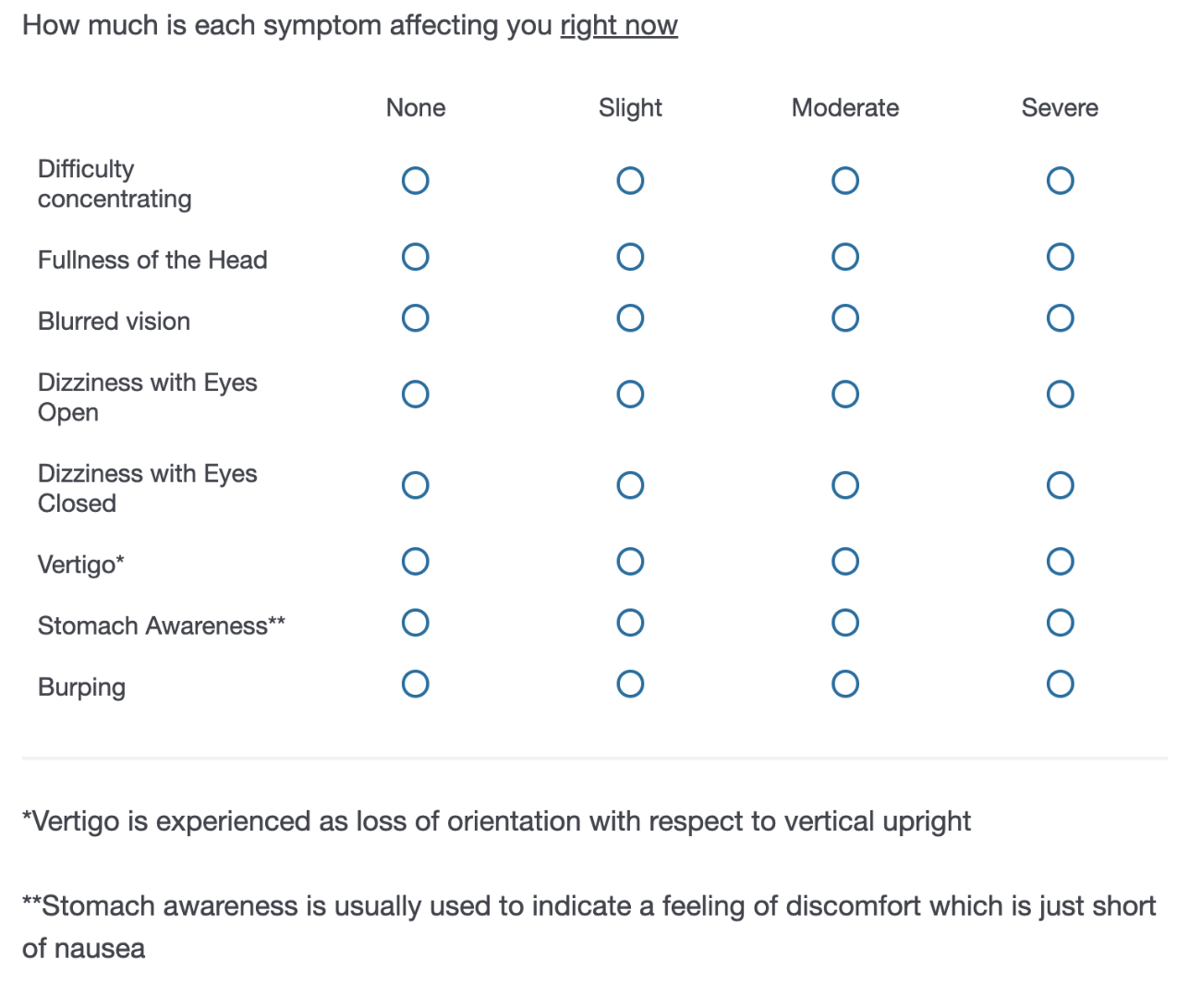


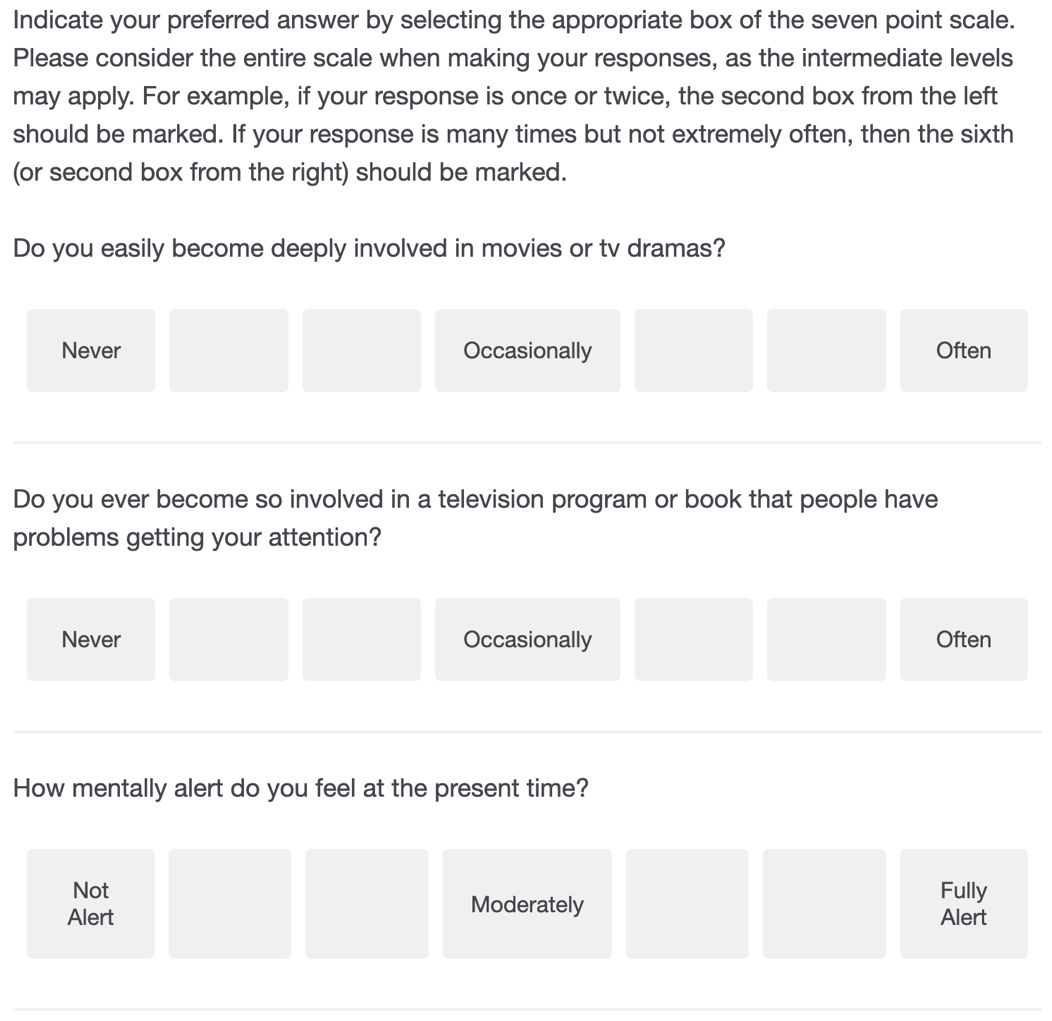


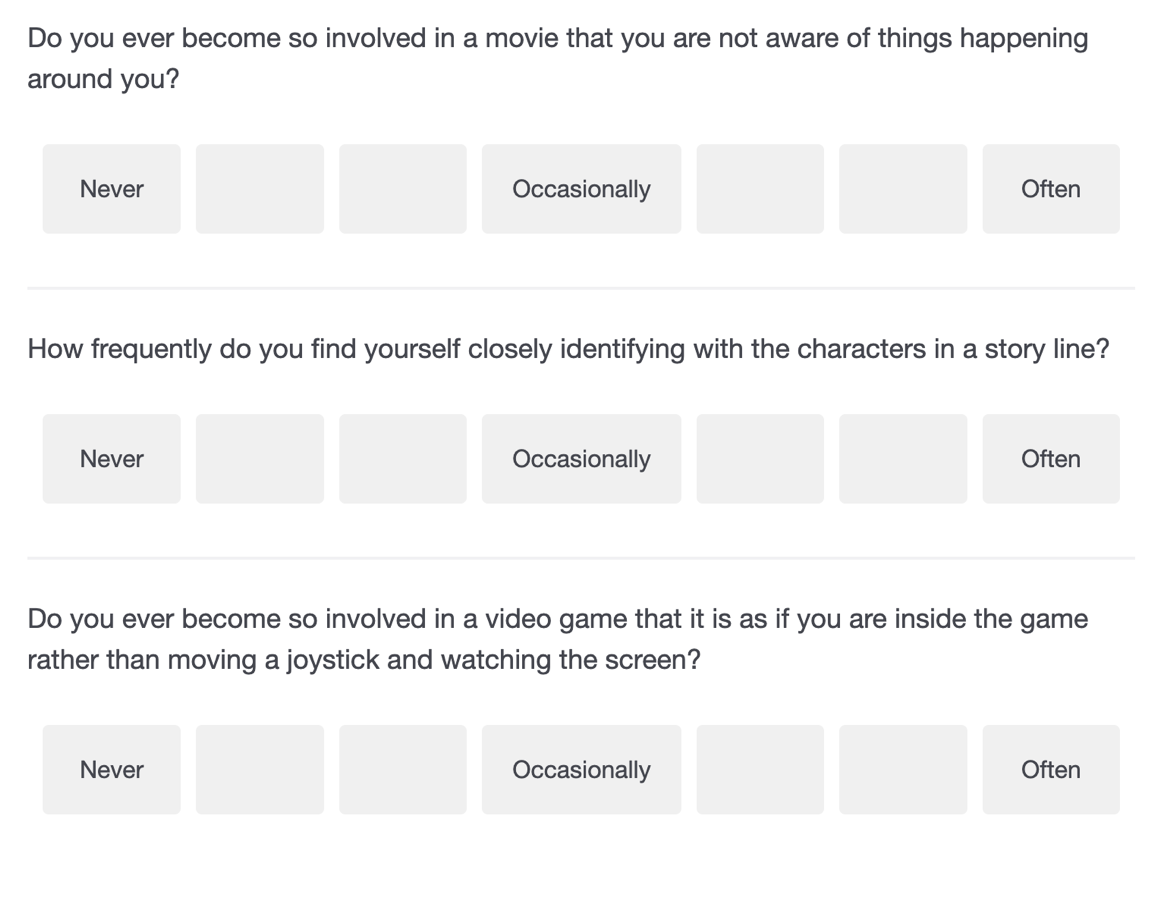


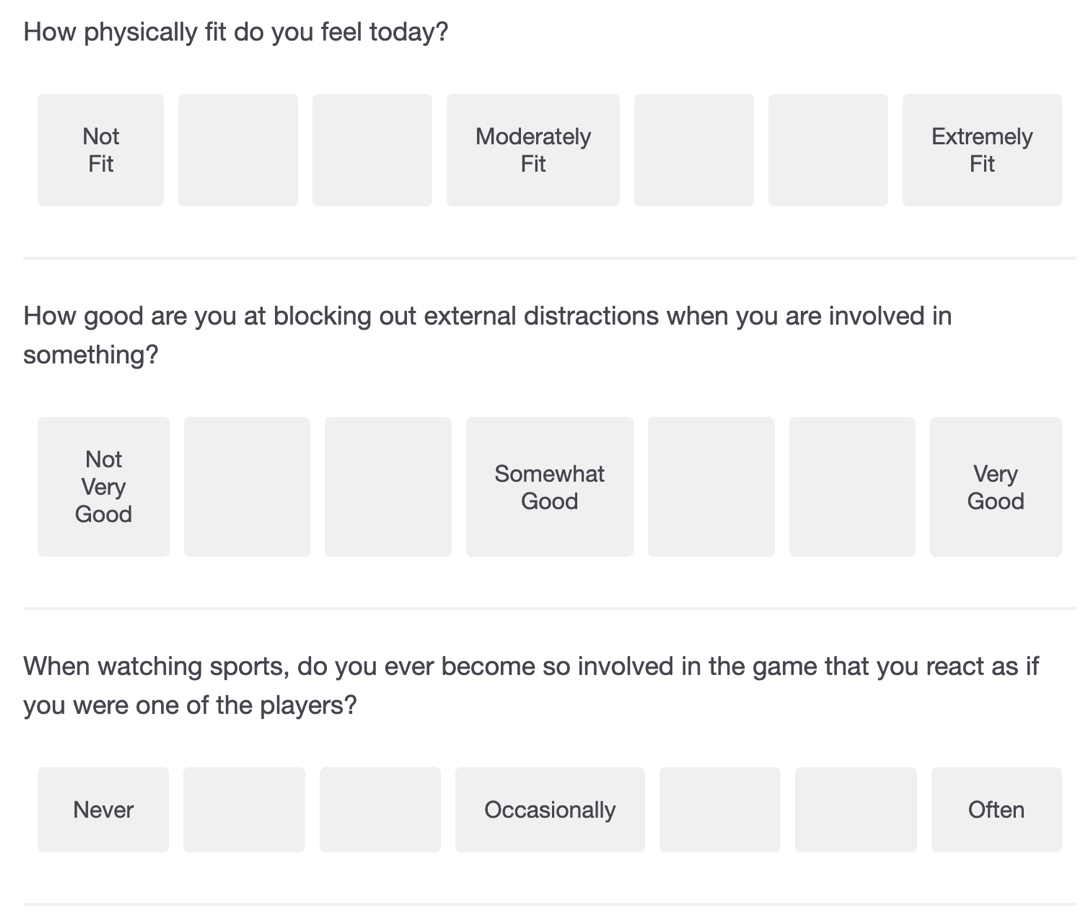


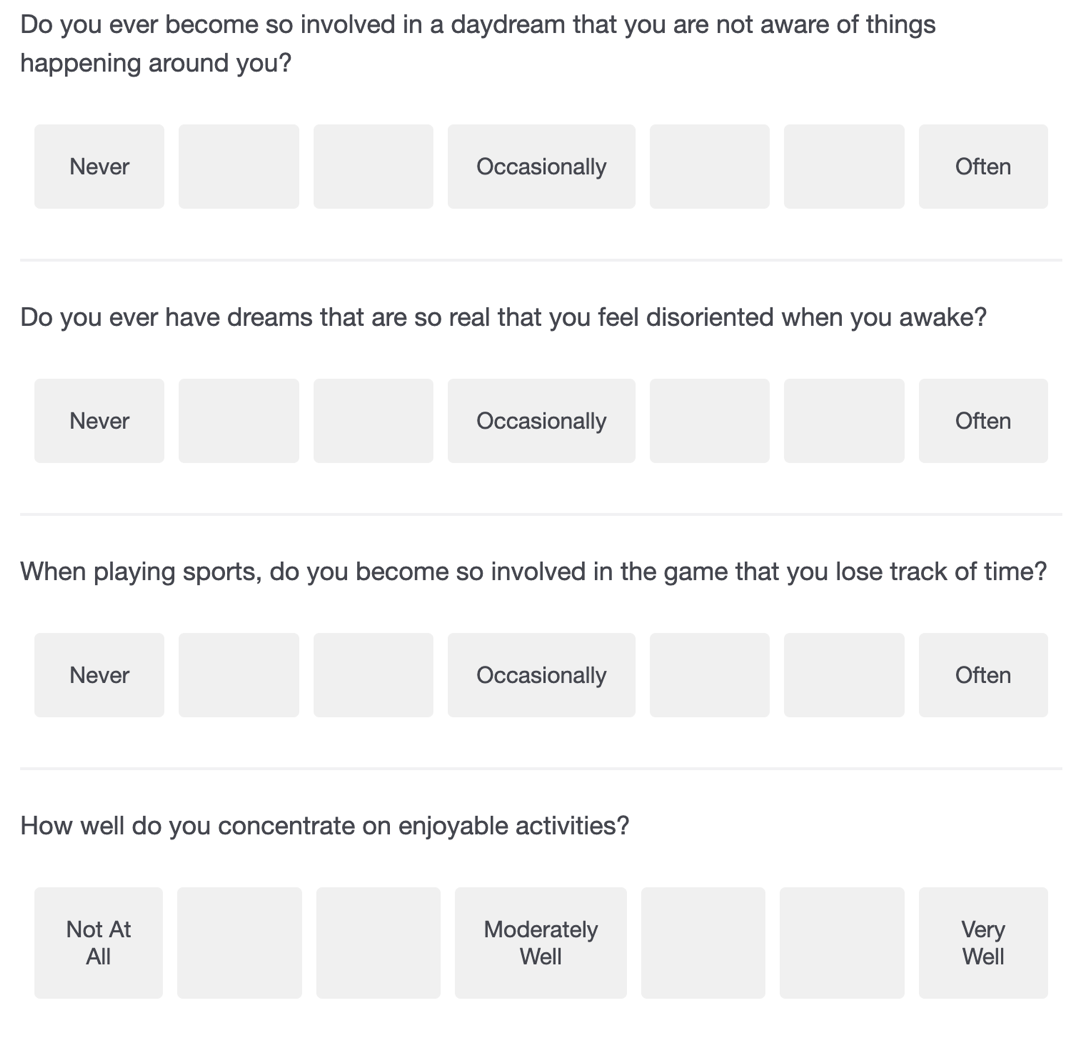


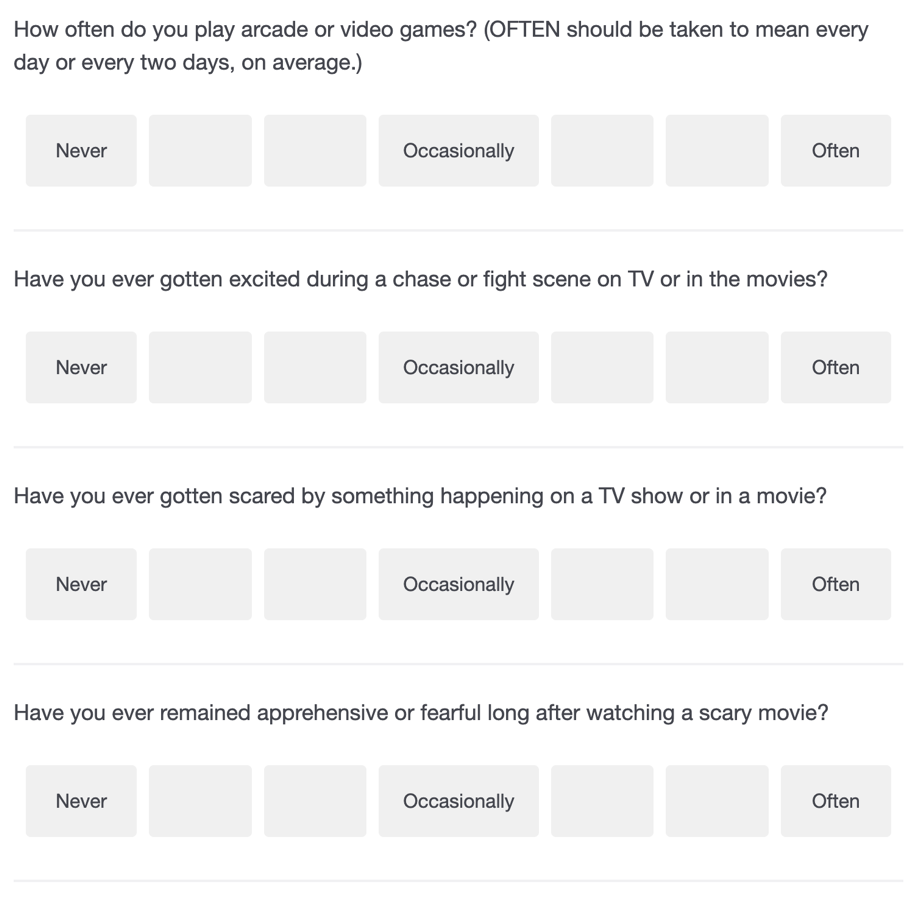


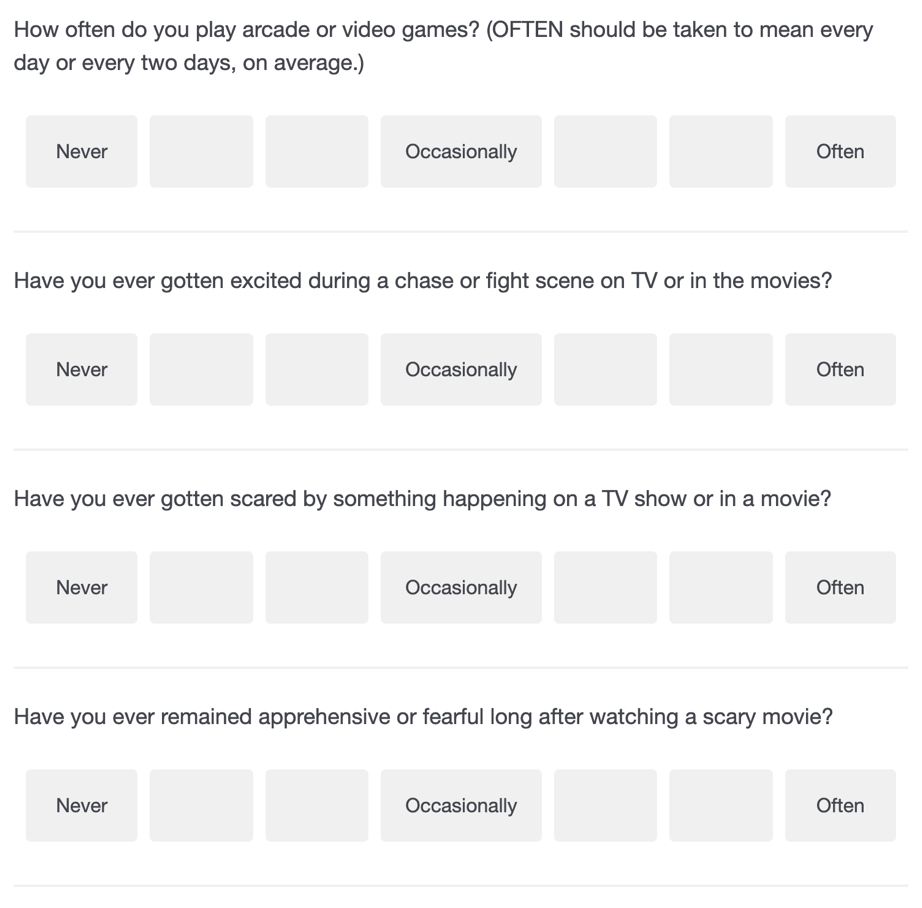


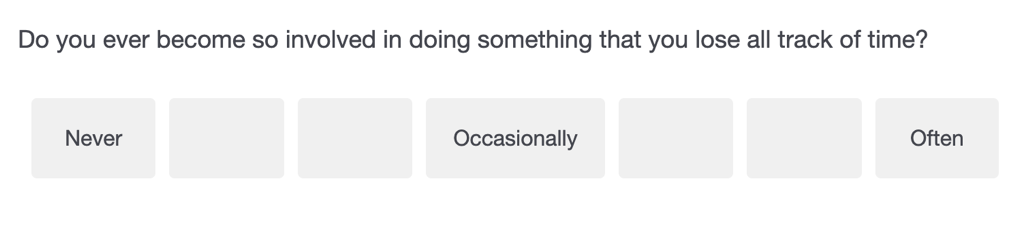


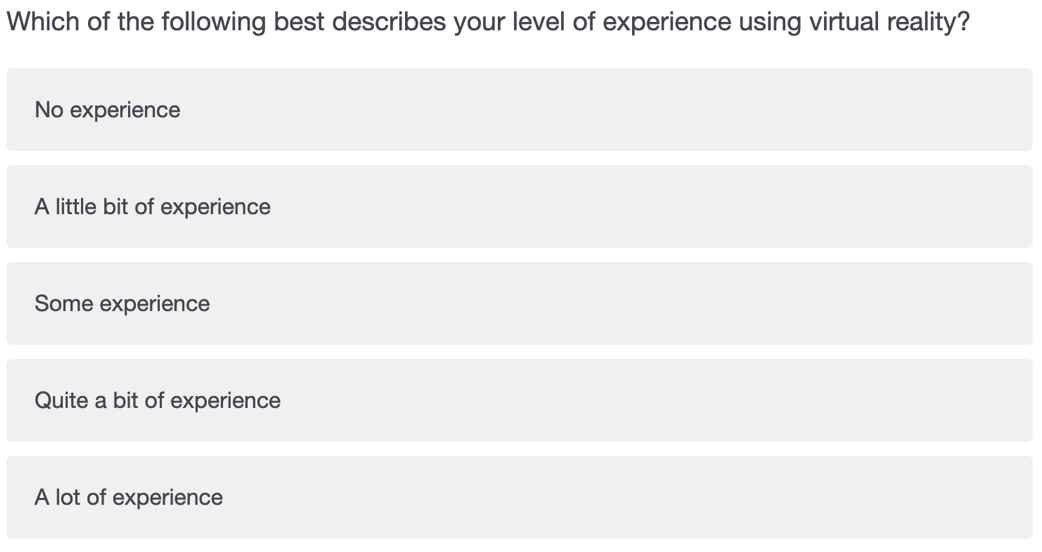


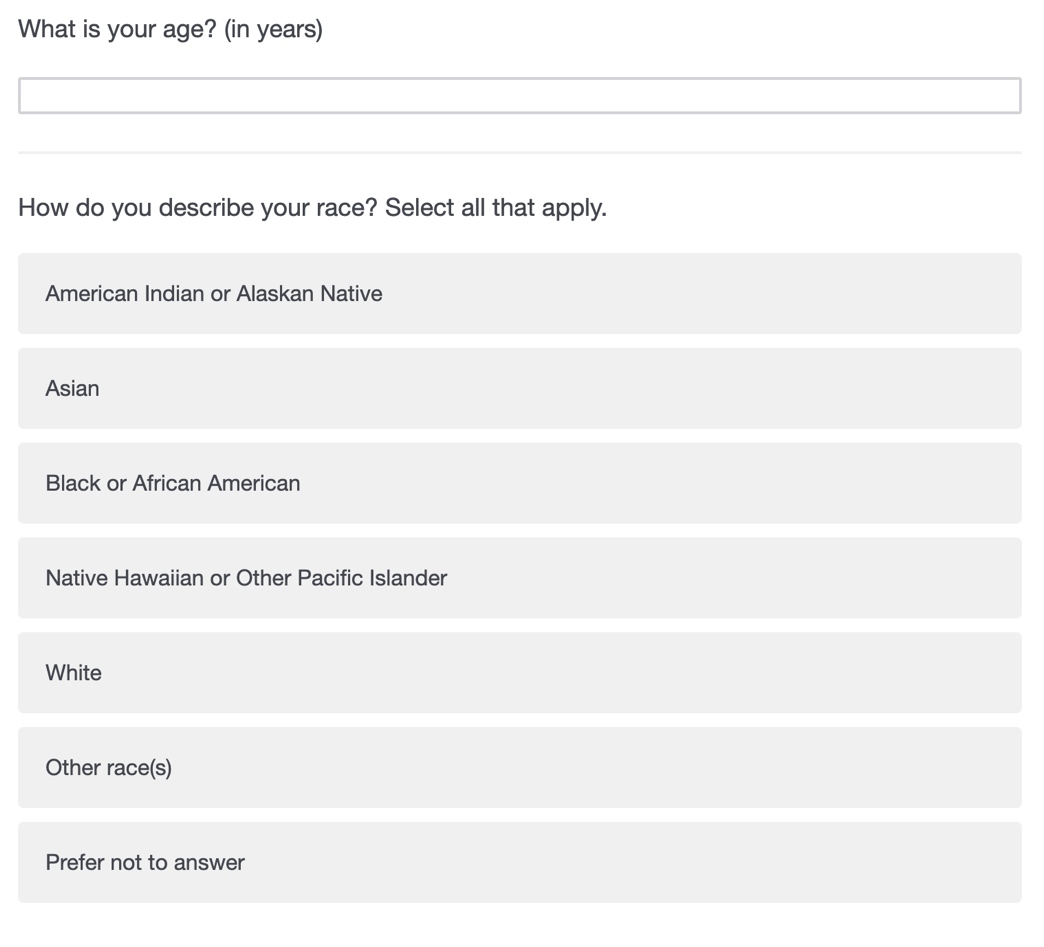


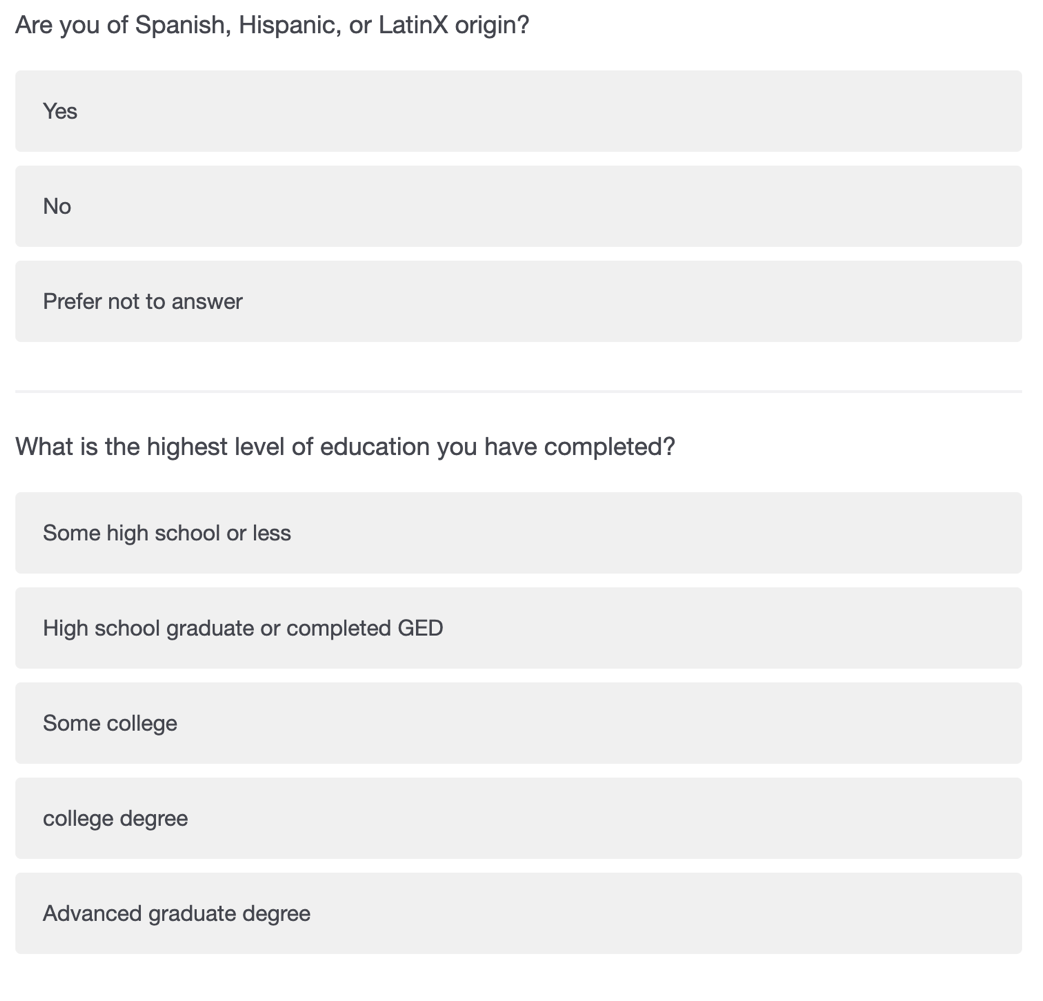


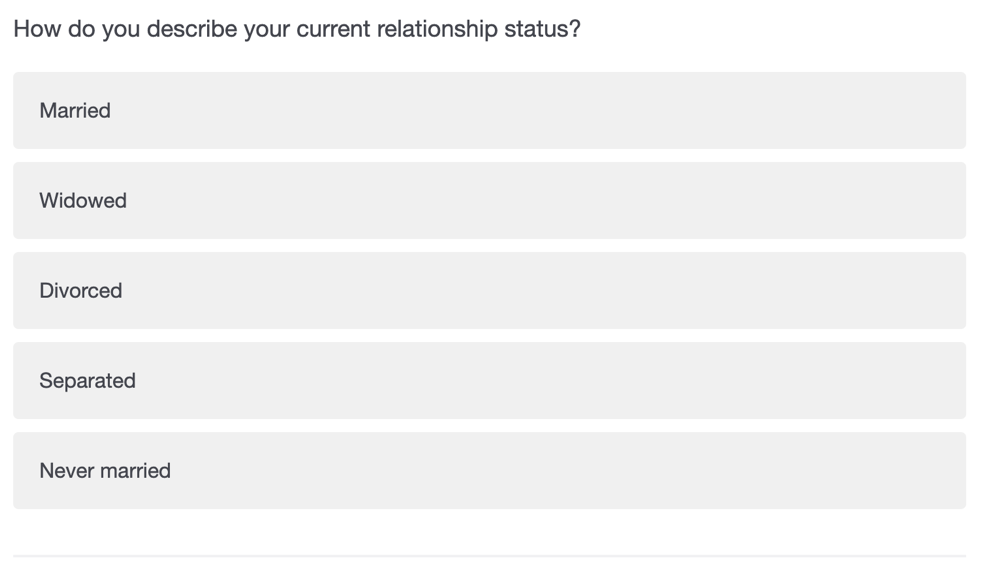


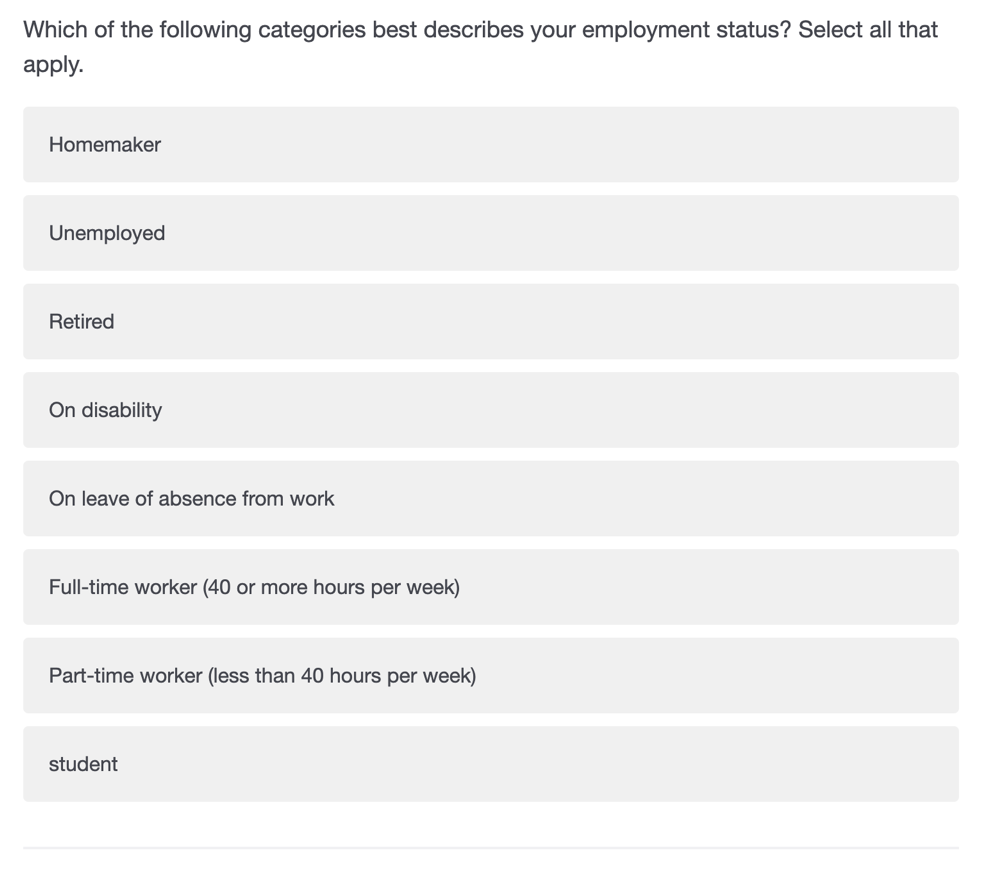


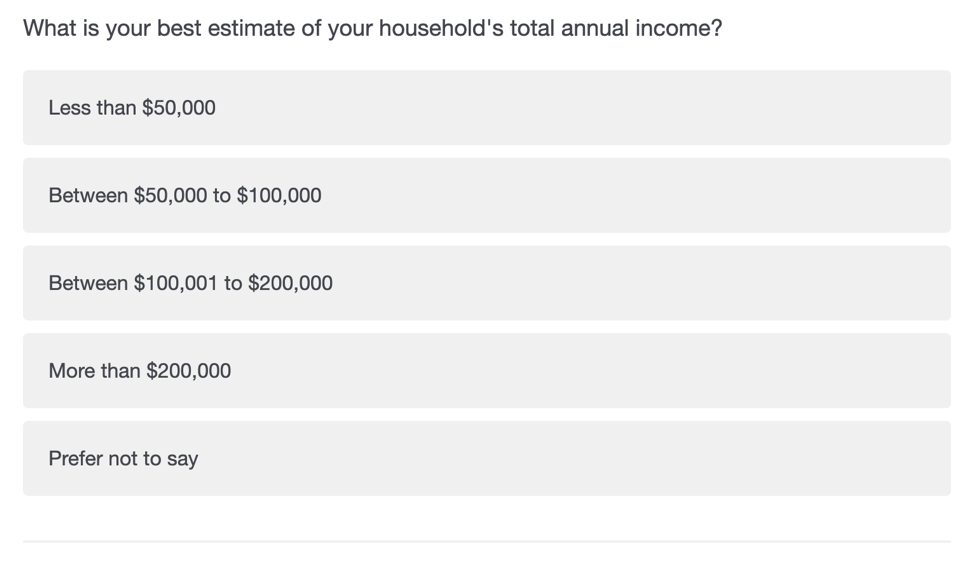


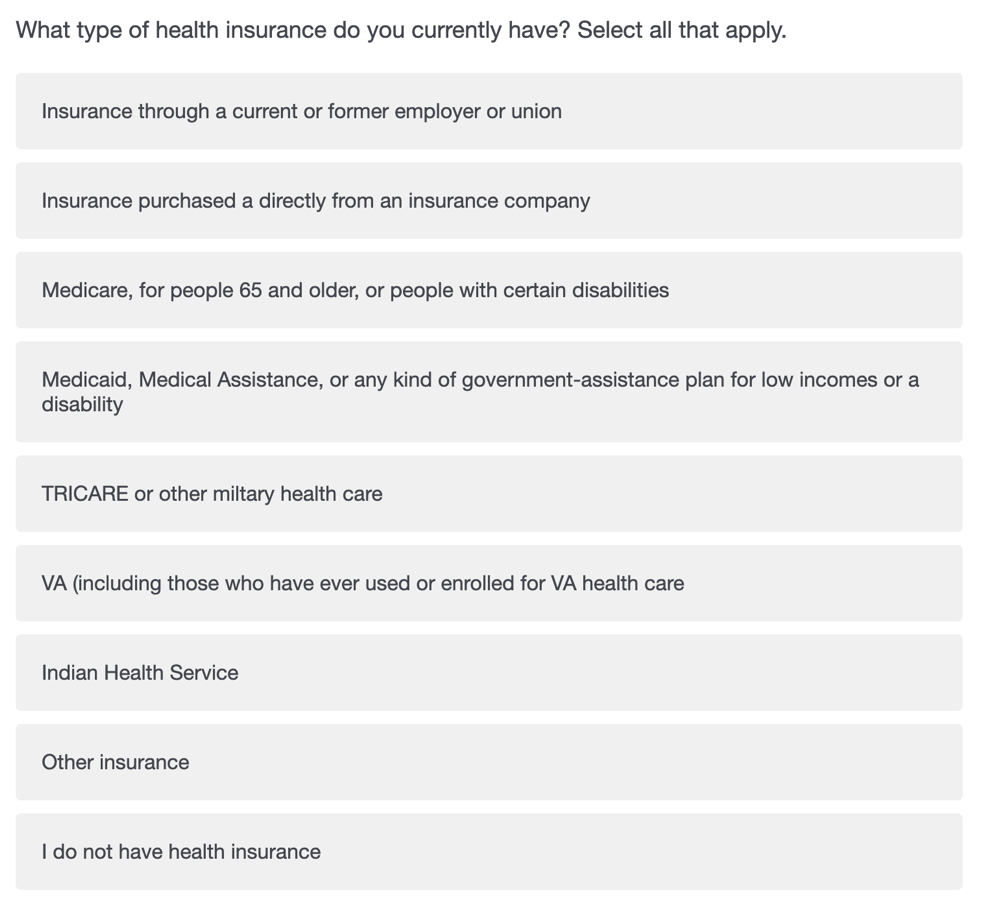


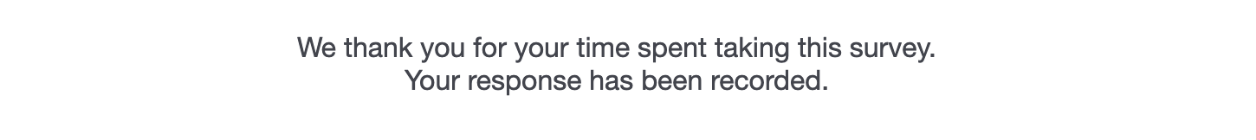


**B. Semi-Structured Interview Guide (10-15 minutes)**

| When you think about what you just experienced, what comes to mind?  What was your favorite part about the experience?  What was your least favorite part of the experience?  What were some barriers to enjoyment for you?  How stressed do you feel now relative to before (when you first arrived)?  Do you think this VR experience would be helpful as a stress reduction technique? If so, please explain how? If not, why?  Would you recommend this experience to a friend or family member?  How could we improve this experience?  What additional content or features could you envision for this experience/app? |
| --- |

**C. Multiple Regression Model for Potential Predictors**

**of Change in STAI-S—Initial Model**

| **Variable** | **Coefficient** | ***P*-value** |
| --- | --- | --- |
| **Overall VR Rating** | -1.9 | .04* |
| **STAI-S**  **Pre-Survey Score** | -0.9 | <.001* |
| **PSS** | 0.09 | .7 |
| **SSQ** | 0.05 | .7 |
| **ITQ** | -0.08 | .6 |
| **Age** | -0.08 | .4 |
| **Gender = Female** | -1.02 | .7 |

**D. Multiple Regression Model for Potential Predictors**

**of Change in STAI-S—Final Model**

| **Variable** | **Coefficient** | ***P*-value** |
| --- | --- | --- |
| **Overall VR Rating** | -2.0 | <.001 |
| **STAI-S**  **Pre-Survey Score** | -0.8 | <.001 |

**E. Longitudinal Statistical Testing Results:**

**Heart Rate (beats per minute)**

| ***PRE-VR***  **Mean** $\boldsymbol{\pm}$ **SD**  **(Median)** | ***POST-VR***  **Mean** $\boldsymbol{\pm}$ **SD**  **(Median)** | | $\boldsymbol{\Delta}$ **(POST-PRE)**  **Mean** $\boldsymbol{\pm}$ **SD**  **(Median)** | **95%CI** | **P-value** |
| --- | --- | --- | --- | --- | --- |
| Baseline  73 $\pm$ 8  (72) | [0-5m] | 68 $\pm$6  (66) | -5 $\pm$ 4  (-4) | [-7.3, -3.1] | <.001 |
|  | [6-10 | 68 $\pm$ 6  (68) | -5 $\pm$ 5  (-4) | [-7.6, -3.0] | <.001 |
|  | [11-15] | 67 $\pm$ 6  (67) | -6 $\pm$ 5  (-5) | [-3.6,- 8.4] | <.001 |
|  | [16-20] | 67 $\pm$ 6  (67) | -6 $\pm$ 5  (-6) | [-8.5, -3.7] | <.001 |
|  | [21-25] | 66 $\pm$ 6  (66) | -6 $\pm$ 5  (-6) | [-9.0, -3.8] | <.001 |
|  | [26-30] | 67 $\pm$ 6  (67) | -6 $\pm$ 6  (-6) | [-9.5, -3.3] | <.001 |
|  | [Post] | 67 $\pm$ 6  (65) | -6 $\pm$ 5  (-5) | [-8.4, -3.5] | <.001 |
| *T, timepoint. SD, standard deviation.* $\boldsymbol{\Delta}$*,* *change.* *CI, confidence interval. m, minute.* | | | | | |

**F. Longitudinal Statistical Testing Results:**

**Time-Domain HRV Parameters**

| **HRV Parameter** | **Pre-VR**  **Mean** $\boldsymbol{\pm}$ **SD**  **(Median)** | **Time** | **Post-VR**  **Mean** $\boldsymbol{\pm}$ **SD**  **(Median)** | $\boldsymbol{\Delta}$ **(POST-PRE)**  **Mean** $\boldsymbol{\pm}$ **SD**  **(Median)** | **95%CI** | ***P*-Value** |
| --- | --- | --- | --- | --- | --- | --- |
| **Root Mean Square of Successive**  **[RR interval] Differences** | Baseline  19.1 $\pm$ 14.7  (13.3) | [0-5m] | 35.3 $\pm$ 46.7  (17.5) | 16.2 $\pm$ 45.0  (1.77) | [-0.066, 18.7] | .054 |
|  |  | [6-10] | 26.4 $\pm$ 22.0  (18.6) | 7.3 $\pm$17.3 (1.21) | [-0.285, 15.4] | .07 |
|  |  | [11-15] | 43.3 $\pm$ 76.1  (19.2) | 24.2 $\pm$76.1  (2.02) | [-0.719, 13.7] | .08 |
|  |  | [16-20] | 30.8 $\pm$ 32.5  (18.8) | 11.7 $\pm$26.6 (2.09) | [0.840, 16.0] | .02* |
|  |  | [21-25] | 30.9 $\pm$  36.8 (19.5) | 11.7 $\pm$28.5 (2.52) | [-0.147, 13.8] | .07 |
|  |  | [26-30] | 30.9 $\pm$ 34.5  (20.4) | 11.1 $\pm$27.2 (3.34) | [-1.85, 16.7] | .07 |
|  |  | [Post] | 25.4 $\pm$ 23.8  (18.2) | 6.33 $\pm$15.1  (3.52) | [0.053, 10.6] | .048* |
| **Standard Deviation of the Interbeat Interval**  **(SDNN)** | Baseline  21 $\pm$13  (18) | [0-5m] | 30$\pm$27  (19) | 8.6 $\pm$25  (1.4) | [-1.9, 12] | .25 |
|  |  | [6-10] | 26 $\pm$18  (20) | 4.3 $\pm$15 (1.8) | [-2.5, 12] | .28 |
|  |  | [11-15] | 37 $\pm$45  (21) | 15 $\pm$44 (1.8) | [-2.2, 14] | .21 |
|  |  | [16-20] | 28$\pm$ 26  (21) | 6.8 $\pm$21 (0.03) | [-2.8, 12] | .47 |
|  |  | [21-25] | 2$9\pm$28  (21) | 7.1 $\pm$ 23 (0.3) | [-3.7, 13] | .47 |
|  |  | [26-30] | 30 $\pm$26  (23) | 8.0 $\pm$ 22  (3.2) | [-3.1, 16] | .19 |
|  |  | [Post] | 2$7\pm$16  (24) | 4.8 $\pm$13  (2.8) | [0.01, 9.2] | .048* |
| *T, timepoint. SD, standard deviation.* $\boldsymbol{\Delta}$*,* *change.* *CI, confidence interval. m, minute.* | | | | | | |

**G. Longitudinal Statistical Testing Results:**

**Frequency-Domain HRV Parameters**

| **HRV Parameter** | **PRE-VR**  **Mean** $\boldsymbol{\pm}$ **SD**  **(Median)** | **Time** | **POST-VR**  **Mean** $\boldsymbol{\pm}$ **SD**  **(Median)** | $\boldsymbol{\Delta}$ **(POST-PRE)**  **Mean** $\boldsymbol{\pm}$ **SD**  **(Median)** | **95%CI** | ***P*-Value** |
| --- | --- | --- | --- | --- | --- | --- |
| **Very Low Frequency** | Baseline  19 $\pm$ 13  (19) | [0-5m] | 10 $\pm$ 7  (11) | -9 $\pm$ 11  (-8) | [-15, -2.6] | .002* |
|  |  | [6-10] | 15 $\pm$ 15  (11) | -4 $\pm$ 16  (-8) | [-10, -0.16] | .048* |
|  |  | [11-15] | 13 $\pm$ 10  (10) | -6 $\pm$ 13  (-9) | [-12, 0.93] | .15 |
|  |  | [16-20] | 13 $\pm$ 11  (10) | -6 $\pm$ 12  (-9) | [-11, -0.16] | .048* |
|  |  | [21-25] | 11 $\pm$ 9  (8) | -8 $\pm$ 15  (-11) | [-14, 0.85] | .07 |
|  |  | [26-30] | 15 $\pm$ 11  (14) | -4 $\pm$ 13  (-5) | [-9.3, 5.2] | .58 |
|  |  | [Post] | 15 $\pm$ 12  (16) | -4 $\pm$ 11  (-3) | [-9.2, 1.0] | .15 |
| **Low Frequency** | 53 $\pm$ 17  (55) | [0-5m] | 50 $\pm$ 23  (48) | -3 $\pm$ 23  (-7) | [-8.9, 15] | .67 |
|  |  | [6-10] | 46 $\pm$ 23  (49) | -6 $\pm$ 23  (-6) | [-4.6, 20] | .30 |
|  |  | [11-15] | $47\pm$ 21  (47) | -6 $\pm$ 20  (-8) | [-3.9, 16] | .27 |
|  |  | [16-20] | 46 $\pm$ 20  (46) | -7 $\pm$ 24  (-9) | [-4.8, 19] | .22 |
|  |  | [21-25] | 49 $\pm$18  (53) | -5 $\pm$ 21  (-2) | [-6.8, 15] | .64 |
|  |  | [26-30] | 48 $\pm18$  (47) | -6 $\pm$ 22  (-7) | [-6.4, 17] | .40 |
|  |  | [Post] | 55 $\pm$ 14  (55) | 1 $\pm$ 19  (0) | [-11, 6.4] | .73 |
| **High Frequency** | 28 $\pm$ 23  (17) | [0-5m] | 40 $\pm$ 26  (32) | 12 $\pm$ 24  (15) | [-24, 1.8] | .10 |
|  |  | [6-10] | 39 $\pm$ 27  (29) | $11\pm$ 19  (12) | [-19, -2.6] | .01* |
|  |  | [11-15] | 40 $\pm$ 27  (32) | 12 $\pm$ 23  (15) | [-23, 0.70] | .07 |
|  |  | [16-20] | 40 $\pm$ 24  (35) | 12 $\pm$ 23  (18) | [-25, -0.40] | .04* |
|  |  | [21-25] | 40 $\pm$ 22  (34) | 12 $\pm$ 19  (17) | [-22, -2.1] | *.02 |
|  |  | [26-30] | $37\pm$ 23  (27) | 8 $\pm$ 21  (10) | [-18, 2.6] | .15 |
|  |  | [Post] | 30 $\pm$ 19  (25) | 2 $\pm$ 19  (8) | [-12.6, 5.9] | .39 |
| **LF:HF Ratio** | 3.$5\pm$ 2.5 (3.5) | [0-5m] | 2.5 $\pm$ 2.6  (1.5) | -1.0 $\pm$ 2.3  (-1.0) | [-2.2 ,0.31] | .12 |
|  |  | [6-10] | $2.2\pm$ 1.8  (1.6) | -1.3 $\pm$ 2.3  (-1.9) | [-2.7,-0.20] | .02* |
|  |  | [11-15] | $3.1\pm$4.2  (1.4) | -0.38 $\pm$ 3.3  (-0.89) | [-2.0, 0.20] | .10 |
|  |  | [16-20] | 2.4$\pm$3.2  (1.4) | -$1.1\pm$ 3.0  (-2.1) | [-2.7, 0.30] | .08 |
|  |  | [21-25] | 2.0 $\pm$1.7  (1.7) | -1.5$\pm$2.1  (-1.8) | [-2.7,-0.40] | .01* |
|  |  | [26-30] | 2.3 $\pm$ 2.0  (2.0) | -1.1 $\pm$2.6  (-1.5) | [-2.4, 0.46] | .10 |
|  |  | [Post] | $2.9\pm$ 2.2  (2.4) | -0.65 $\pm$2.7  (-1.1) | [-2.1, 0.85] | .47 |
| *T, timepoint. SD, standard deviation.* $\boldsymbol{\Delta}$*,* *change.* *CI, confidence interval. m, minute.* | | | | | | |

**H. Additional Survey Results:**

**Participants Experience with Stress Reduction Methods**

|  | **Never** | **Tried once or twice**  **(or tried in past but not currently)** | **A few times/rarely**  **(<1x/month)** | **Sometimes (1x/month)** | **Frequently (weekly or daily)** |
| --- | --- | --- | --- | --- | --- |
| **Meditation** | 5 | 7 | 2 | 2 | 4 |
| **Mindfulness** | 6 | 7 | 1 | 1 | 5 |
| **Yoga** | 5 | 11 | 2 | 2 | 0 |
| **Deep Breathing** | 5 | 3 | 4 | 3 | 5 |
| **Massage** | 5 | 7 | 3 | 4 | 1 |
| **Tai Chi** | 8 | 11 | 0 | 1 | 0 |
| **Biofeedback** | 10 | 8 | 2 | 0 | 0 |
| **Progressive Muscle Relaxation** | 9 | 10 | 0 | 1 | 0 |
| **Music and Art Therapy** | 9 | 6 | 3 | 1 | 1 |
| **Aromatherapy** | 7 | 7 | 2 | 2 | 2 |
| **Hydrotherapy** | 9 | 10 | 0 | 0 | 1 |
| **Exercise** | 3 | 3 | 1 | 0 | 13 |
| **Other: Journaling x1, Psychotherapy x1** | | | | | |

**I. Additional Survey Results:**

**Participants Experience with Exercise, Caffeine, & Sleep**

| Exercise Today? | Yes = 9  No = 11 |
| --- | --- |
| Exercise Regularly? | Yes = 14  No = 6 |
| How many caffeinated beverages did you have today? (cups of coffee, cans of soda or energy drinks) | None (or less than 1 serving/decaf) = 9  1-2 servings = 10  3-4 servings = 1 |
| How many caffeinated beverages do you generally have on a daily basis? (cups of coffee, cans of soda or energy drinks) | None (or less than 1 serving/decaf) = 6  1-2 servings = 11  3-4 servings = 2  4-6 servings = 1 |
| How long ago did you last have a caffeinated beverage? | I don’t consume caffeine = 5  Within last hour = 4  1 to 4 hours ago = 7  > 12 hours ago = 4 |
| About how many hours did you sleep last night? | Less than 5 hours = 5  5 to 6 hours = 4  6 to 7 hours = 8  7 to 8 hours = 3 |
| GENERALLY speaking, about how many hours do your sleep per night? | Less than 5 hours = 2  5 to 6 hours = 5  6 to 7 hours = 8  7 to 8 hours = 4  9 or more hours = 1 |
